# Neonatal, infant, and childhood growth following metformin versus insulin treatment for gestational diabetes: A systematic review and meta-analysis

**DOI:** 10.1371/journal.pmed.1002848

**Published:** 2019-08-06

**Authors:** Jane L. Tarry-Adkins, Catherine E. Aiken, Susan E. Ozanne

**Affiliations:** 1 Metabolic Research Laboratories and MRC Metabolic Diseases Unit, Wellcome Trust–MRC Institute of Metabolic Science, University of Cambridge, Cambridge, United Kingdom; 2 Department of Obstetrics and Gynaecology, Rosie Hospital and NIHR Cambridge Comprehensive Biomedical Research Centre, University of Cambridge, Cambridge, United Kingdom; Peking Univeristy First Hospital, CHINA

## Abstract

**Background:**

Metformin is increasingly offered as an acceptable and economic alternative to insulin for treatment of gestational diabetes mellitus (GDM) in many countries. However, the impact of maternal metformin treatment on the trajectory of fetal, infant, and childhood growth is unknown.

**Methods and findings:**

PubMed, Ovid Embase, Medline, Web of Science, ClinicalTrials.gov, and the Cochrane database were systematically searched (from database inception to 26 February 2019). Outcomes of GDM-affected pregnancies randomised to treatment with metformin versus insulin were included (randomised controlled trials and prospective randomised controlled studies) from cohorts including European, American, Asian, Australian, and African women. Studies including pregnant women with pre-existing diabetes or non-diabetic women were excluded, as were trials comparing metformin treatment with oral glucose-lowering agents other than insulin. Two reviewers independently assessed articles for eligibility and risk of bias, and conflicts were resolved by a third reviewer. Outcome measures were parameters of fetal, infant, and childhood growth, including weight, height, BMI, and body composition. In total, 28 studies (*n =* 3,976 participants) met eligibility criteria and were included in the meta-analysis. No studies reported fetal growth parameters; 19 studies (*n =* 3,723 neonates) reported measures of neonatal growth. Neonates born to metformin-treated mothers had lower birth weights (mean difference −107.7 g, 95% CI −182.3 to −32.7, *I*_2_ = 83%, *p =* 0.005) and lower ponderal indices (mean difference −0.13 kg/m^3^, 95% CI −0.26 to 0.00, *I*_2_ = 0%, *p =* 0.04) than neonates of insulin-treated mothers. The odds of macrosomia (odds ratio [OR] 0.59, 95% CI 0.46 to 0.77, *p <* 0.001) and large for gestational age (OR 0.78, 95% CI 0.62 to 0.99, *p =* 0.04) were lower following maternal treatment with metformin compared to insulin. There was no difference in neonatal height or incidence of small for gestational age between groups. Two studies (*n =* 411 infants) reported measures of infant growth (18–24 months of age). In contrast to the neonatal phase, metformin-exposed infants were significantly heavier than those in the insulin-exposed group (mean difference 440 g, 95% CI 50 to 830, *I*_2_ = 4%, *p =* 0.03). Three studies (*n =* 520 children) reported mid-childhood growth parameters (5–9 years). In mid-childhood, BMI was significantly higher (mean difference 0.78 kg/m^2^, 95% CI 0.23 to 1.33, *I*_2_ = 7%, *p =* 0.005) following metformin exposure than following insulin exposure, although the difference in absolute weights between the groups was not significantly different (*p =* 0.09). Limited evidence (1 study with data treated as 2 cohorts) suggested that adiposity indices (abdominal [*p =* 0.02] and visceral [*p =* 0.03] fat volumes) may be higher in children born to metformin-treated compared to insulin-treated mothers. Study limitations include heterogeneity in metformin dosing, heterogeneity in diagnostic criteria for GDM, and the scarcity of reporting of childhood outcomes.

**Conclusions:**

Following intrauterine exposure to metformin for treatment of maternal GDM, neonates are significantly smaller than neonates whose mothers were treated with insulin during pregnancy. Despite lower average birth weight, metformin-exposed children appear to experience accelerated postnatal growth, resulting in heavier infants and higher BMI by mid-childhood compared to children whose mothers were treated with insulin. Such patterns of low birth weight and postnatal catch-up growth have been reported to be associated with adverse long-term cardio-metabolic outcomes. This suggests a need for further studies examining longitudinal perinatal and childhood outcomes following intrauterine metformin exposure. This review protocol was registered with PROSPERO under registration number CRD42018117503.

## Abstract 2

Jane L. Tarry-Adkins and colleagues report that neonates whose mothers were treated for gestational diabetes with metformin as opposed to insulin weigh less at birth but have a higher BMI by mid-childhood.

## Author summary

**Why was this study done?:**

- Gestational diabetes is a common pregnancy complication, affecting around 1 in 7 pregnancies worldwide, and can be associated with adverse outcomes for both mothers and babies.
- There are several effective treatment strategies available for gestational diabetes, including using metformin or insulin to control high blood sugars in the mother.
- It is important to fully understand any effects that these 2 treatment options may have on the growth of the baby in the womb and also after birth into childhood.

**What did the researchers do and find?:**

- We performed a systematic review of 28 studies that included 3,976 mothers who were randomised to metformin or insulin for treatment of gestational diabetes. We included all studies that reported the weight and growth of their babies in the womb, at birth, or later in childhood.
- We showed that babies whose mothers were treated with metformin weighed on average 108 g less at birth than those whose mothers were treated with insulin, and had a lower risk of being born large (>90th centile) for their gestation at delivery.
- Metformin-exposed infants were 0.44 kg heavier by 18–24 months than insulin-exposed infants. Metformin-exposed children had higher BMI (by 0.8 kg/m^2^) by mid-childhood (5–9 years) than insulin-exposed children.

**What do these findings mean?:**

- Metformin treatment for gestational diabetes alters the postnatal growth trajectory compared to insulin treatment. Children exposed to metformin compared to insulin in the womb tend to be born at significantly lower birth weights, but are heavier in infancy, with higher BMI by mid-childhood.
- It is known that children who are born small and then undergo ‘catch-up growth’ after birth are at increased risk of developing cardiovascular disease and type 2 diabetes later in life. It is important to understand whether this increased risk applies to children whose mothers were treated with metformin during pregnancy.

## Introduction

Gestational diabetes mellitus (GDM) currently affects 3%–25% of pregnancies worldwide [1], constituting a significant global healthcare burden. The continuing increase in the global incidence of GDM may relate to new screening approaches, decreasing threshold values for diagnosis, or increases in population risk factors, particularly obesity [2,3]. GDM poses significant risks to the immediate and long-term health of the mother and fetus. For the fetus, a key risk is accelerated intrauterine growth, resulting in macrosomic or large for gestational age (LGA) neonates [4,5]. At delivery, macrosomic and LGA neonates are at significantly elevated risk of adverse perinatal outcomes, including shoulder dystocia, birth trauma, neonatal hypoglycaemia, and admission to neonatal intensive care [6,7]. Hence it is essential to implement effective clinical interventions to maintain glycaemic control and limit fetal growth to within normal parameters during GDM-affected pregnancies [8].

Insulin, the main endogenous hormone responsible for maintaining glucose homeostasis, is an effective treatment for GDM [9]. However, the efficacy of insulin is weighed against significant disadvantages. Insulin use can provoke maternal hypoglycaemia [10], increases the tendency to maternal weight gain [11], requires injection, and is cumbersome to administer and monitor [12,13]. In particular, the expense of insulin therapy and the difficulties with refrigerated storage make it less suitable for use in low and middle development index settings, where some of the greatest increases in incidence of GDM are currently observed [14]. Therefore, the possibility of using oral glucose-lowering agents for treatment of GDM has been an area of intense research interest [15–17].

Metformin (N,N-dimethylbiguanide), a biguanide oral glucose-lowering drug, has gained widespread acceptance. Metformin is approved for use in the treatment of GDM in many countries across the world and is featured in the 20th World Health Organization (WHO) essential medicines list [18], and it has been recommended by the Society for Maternal–Fetal Medicine (SMFM) as a first line treatment for GDM [19]. Available evidence suggests that metformin is effective in maintaining maternal glycaemic control and may help to limit gestational weight gain ([20] and reviewed in [21]). However, unlike insulin, metformin crosses the placenta and is present at clinically relevant concentrations in fetal and placental tissues (50%–100% of maternal concentrations) [22,23]. Thus, metformin exposure could potentially affect the developing feto-placental unit via direct or indirect pathways other than control of maternal hyperglycaemia.

A key aim of GDM treatment is to prevent fetal over-growth; consequently, several randomised trials have examined the impact of metformin treatment on birth weight in GDM-affected pregnancies [15–17,24–32]. However, there is no clear consensus on how fetal growth is affected by metformin treatment, and whether intrauterine exposure to metformin has longer-term consequences for growth in infancy and childhood. The aim of our study was therefore to determine whether metformin compared to insulin treatment for GDM alters perinatal growth trajectories or body composition from the time of fetal exposure through to mid-childhood. Addressing this issue is particularly important as the number of pregnancies exposed to metformin increases worldwide [8].

## Methods

This systematic review and meta-analysis was conducted in accordance with the Preferred Reporting Items for Systematic Reviews and Meta-Analyses (PRISMA) guidelines [33]. The PRISMA checklist is detailed in S1 PRISMA Checklist. The systematic review protocol was registered in PROSPERO (CRD42018117503) (S1 Text). Ethical approval was not required.

### Literature searches, search strategies, and eligibility criteria

Systematic literature searches using pre-specified terms (S2 Text) were performed on PubMed (June 1997 to 26 February 2019), Ovid Embase (1974 to 26 February 2019), Ovid Medline (1946 to 26 February 2019), Cochrane Library (database inception to 26 February 2019), ClinicalTrials.gov (database inception to 26 February 2019), and Web of Science (1900 to 26 February 2019). No filters were applied to any of the searches. No language or location restrictions were applied.

Studies that randomised women with GDM to metformin versus insulin therapy were included. Studies were excluded if they compared metformin to other oral glucose-lowering agents (e.g., glyburide) or if interventions were given prior to pregnancy. GDM was screened for and diagnosed according to local criteria in each study, and we did not apply exclusions with respect to this. Studies were excluded if they included participants with multiple pregnancies or pre-existing diabetes, or if they randomised fewer than 50 women in total. Studies were excluded if trial participants were omitted from the study on the basis of fetal weight and/or birth weight. Data reported only in meeting abstracts would have been included if the abstract contained sufficient information for assessment, but none met this standard. Where the available information for assessment was insufficient, authors were contacted for further information. Two out of the 8 authors contacted for further information responded to this request. One author reported the use of permuted block randomisation in their study [34], and the other stated that a random allocation of treatments was used, yet did not specify what the nature of the randomisation was, and that study assessors were blinded to the treatment groups [25].

### Study selection and data extraction

**Fig 1:**
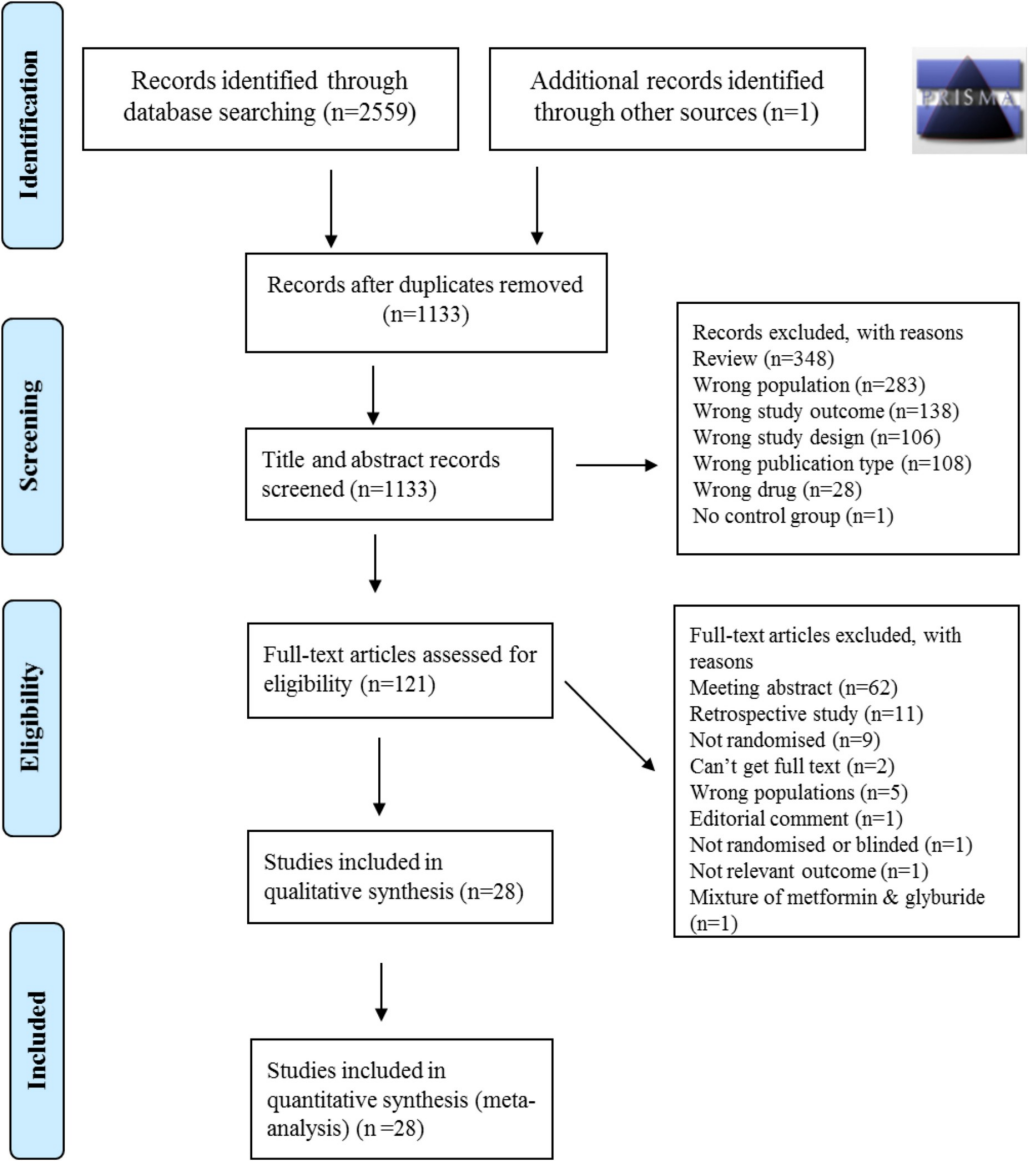
PRISMA flow diagram.

Two reviewers (JLT-A and CEA) independently assessed each study using predetermined inclusion/exclusion criteria (detailed in S1 Table). A third reviewer (SEO) was available to resolve cases where eligibility was unclear. An initial screen of titles and abstracts was performed, followed by a detailed full paper screen. The results from each step of the review process are documented in a PRISMA flow diagram (Fig 1).

Data extraction from eligible studies was conducted independently by 2 authors (JLT-A and CEA). Outcome measures were fetal growth parameters (head circumference, abdominal circumference, femur length, biparietal diameter, and estimated fetal weight calculated by any formula; millimetres or grams); birth weight (grams or kilograms); small for gestational age (SGA) (birth weight < 10th centile for gestational age; *n* and percent); LGA (birth weight > 90th centile for gestational age; *n* and percent); macrosomia (birth weight > 4 kg; *n* and percent); neonatal, infant, and child heights (centimetres); neonatal ponderal index (kg/m^3^); BMI (kg/m^2^); abdominal, head, chest, and waist circumferences (centimetres); skinfold thicknesses (bicep, tricep, and subscapular; millimetres); fat masses (arm, abdominal, thigh, and total; grams); and fat volumes (abdominal, visceral, and abdominal subcutaneous; cm^3^). Where multiple time points were reported for the same population, we included data from each time point, provided it fell within 1 of the 4 specified study time frames: (i) third trimester of pregnancy (28–40 weeks), (ii) neonatal period (at birth or within 6 weeks), (iii) infancy (18 months–2 years), or (iv) mid-childhood (5–9 years).

### Quality assessment of included studies (risk of bias in individual studies)

Each study was independently assessed by 2 authors (JLT-A and CEA) for quality and validity using the Cochrane Collaboration tool for assessing risk of bias. Seven risk-of-bias domains were assessed for each study, and each domain was given a rating of low risk, unknown risk, or high risk of bias (S2 Table). All risk-of-bias analysis was conducted at the study level.

The principle summary measures utilised in this systematic review were unadjusted odds ratios (ORs) (for dichotomous data) or differences in means (for continuous data). Meta-analysis was performed using Review Manager (RevMan) version 5.3 (Nordic Cochrane Centre, Copenhagen, 2014) and the ‘*metafor*’ package in R version 3.5.1 [35]. Funnel plots were constructed to assess publication bias, and outcome measures with 5 or more studies included were also subjected to Egger’s test. Heterogeneity between studies was assessed using the *I*^2^ statistic, and any outcomes showing significant inter-study heterogeneity were analysed using a random-effects model. Sensitivity analyses were performed using leave-one-out testing for individual studies and for studies grouped according to GDM criteria [35], and by conducting meta-analyses using only the subset of studies assessed as being high quality (i.e., low risk of bias). Where *p-*values are reported, an alpha level < 0.05 was considered statistically significant.

## Results

### Study selection

Electronic searching of the specified databases yielded a total of 2,559 studies, and 1 further study was found via hand-searching. After removal of duplicates and title/abstract screening, 121 trials received full text assessment, applying the full set of eligibility criteria (Fig 1). After full text evaluation, a total of 28 studies remained eligible for inclusion, representing 3,976 pregnancies (Fig 1).

The included studies varied in terms of quality and design (S3 Table). Outcomes measured varied between studies, with birth weight the most commonly reported outcome (17 studies). There was considerable clinical heterogeneity in the dose of metformin used (ranging from 500 mg to 3,000 mg daily) both within and between studies. There was also heterogeneity between studies in criteria used to diagnose GDM, with a total of 8 different diagnostic criteria used, including those specified by the American College of Obstetricians and Gynecologists, American Diabetes Association, Australasian Diabetes in Pregnancy Society, Carpenter–Coustan, Finnish national criteria, International Association of Diabetes and Pregnancy Study Groups, National Diabetes Data Group, and WHO (S3 Table). We therefore performed a leave-one-criterion-out sensitivity analysis for birth weight (the primary and best-evidenced finding of this meta-analysis) to demonstrate that use of different thresholds for GDM diagnosis did not have a significant impact on the result (S1 Fig). There was also a range of geographical settings including Europe [15,17,36–38], US [39], Australia/New Zealand [16,40,41], South Asia [31,42] and Africa and the Middle East [24–30,34,43–46].

The risk of bias was low in the majority of included studies. However, 6 studies did not analyse data on an intention-to-treat basis (i.e., trial participants who did not achieve adequate glycaemic control with metformin were removed from the study [24–26,28,30,34], leading to an overall high risk of bias). A further study had significant imbalance in the baseline characteristics of participants, potentially due to failure of randomisation [42]. We performed subgroup meta-analyses excluding the studies assessed as having an overall high risk of bias (low-quality studies). Subgroup analysis of only high-quality studies showed that removal of the low-quality studies did not materially alter the outcome of the meta-analysis for any of the outcomes assessed; therefore, all studies were included (S2 Fig). We also assessed the likelihood of single studies significantly influencing the overall results using leave-one-out analysis. We demonstrated that the results of the meta-analyses of birth weight, macrosomia, and SGA were robust to the omission of single studies. The summary effect size for the outcome of LGA was non-significant when various studies were removed, decreasing our confidence in the robustness of this finding (S3 Fig). Funnel plots for all outcomes were assessed visually (S4 Fig). There were no obvious asymmetries in the plots for any study outcomes, with the exception of macrosomia in studies judged to be high quality. Egger’s test (*p <* 0.05) confirmed the likelihood of publication bias with respect to macrosomia in these studies, but not with respect to all included studies. Visual inspection of the funnel plots confirmed that there was no reason to believe that significant publication bias affected any of the other outcomes studied.

### Fetal growth and neonatal outcomes

No eligible studies reported fetal growth outcomes in metformin- versus insulin-treated GDM pregnancies.

**Fig 2:**
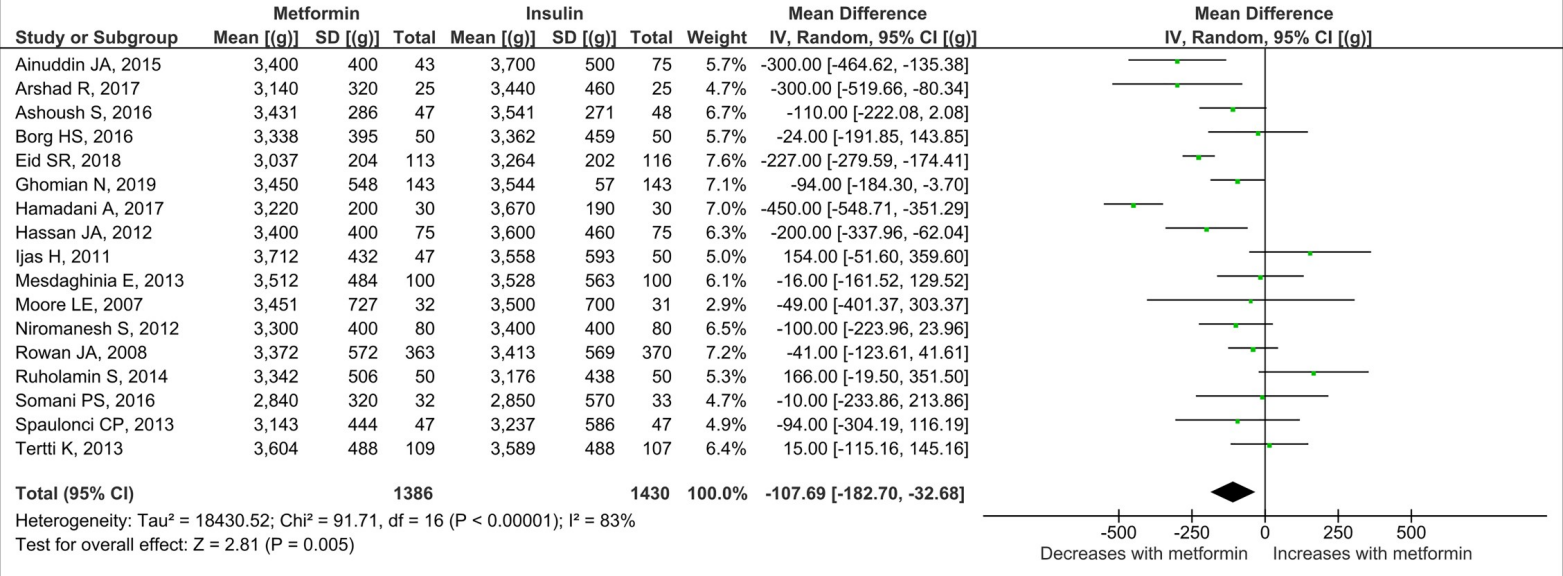
Birth weight. Expressed as mean difference (random effects model). IV, mean difference.

**Fig 3:**
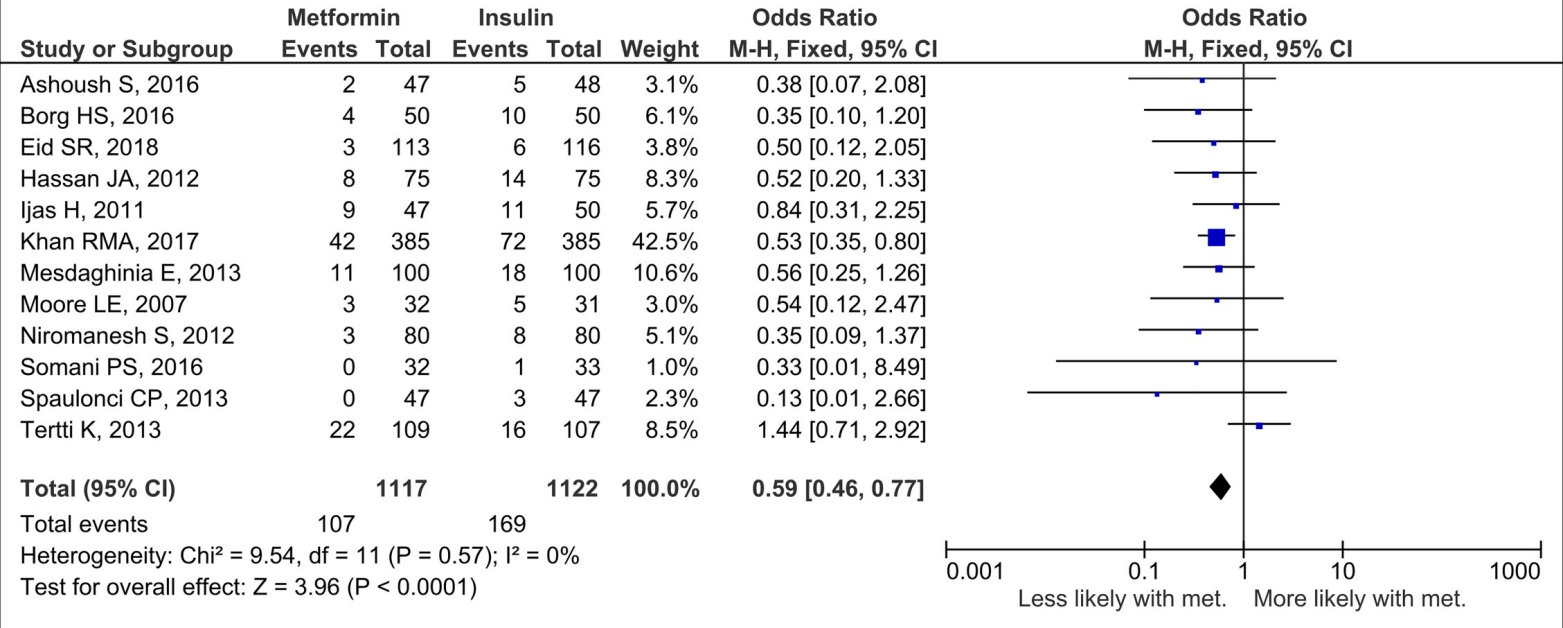
Macrosomia. Expressed as OR (fixed effects model) and 95% CI. M-H, odds ratio; met., metformin.

**Fig 4:**
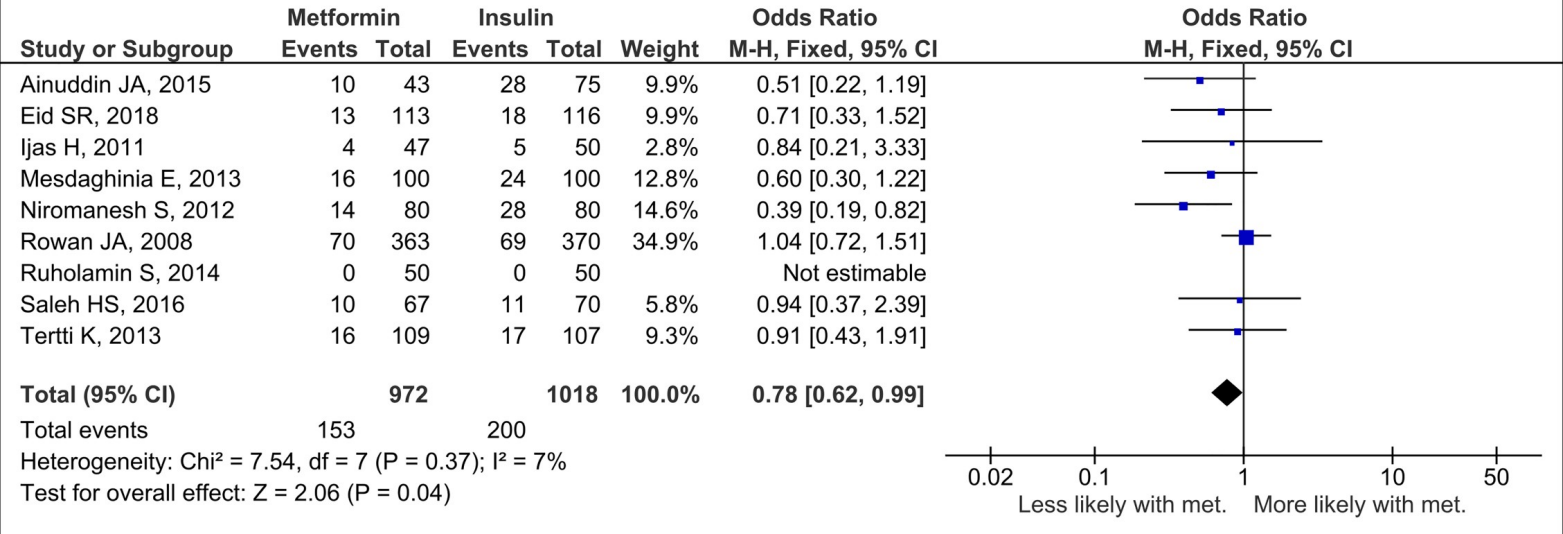
Large for gestational age. Expressed as OR (fixed effects model) and 95% CI. M-H, odds ratio; met., metformin.

**Fig 5:**
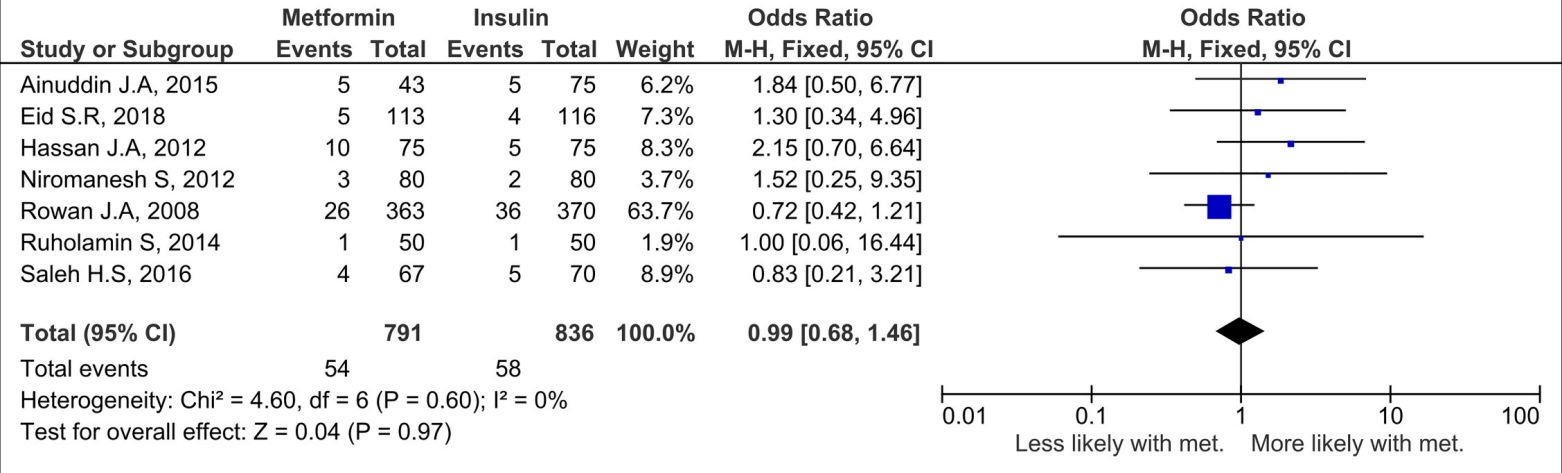
Small for gestational age. Expressed as OR (fixed effects model) and 95% CI. M-H, odds ratio; met., metformin.

**Fig 6:**
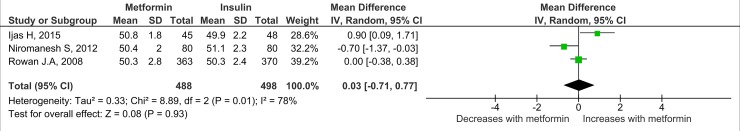
Neonatal height. Expressed as mean difference (random effects model) and 95% CI. IV, mean difference.

Nineteen studies (*n =* 3,723 neonates) reported neonatal growth parameters. Birth weights of neonates born to mothers treated with metformin were significantly lower than those of neonates whose mothers were treated with insulin during pregnancy. On average, metformin-exposed neonates were 107.7 g smaller than those whose mothers were randomised to insulin (95% CI 32.7 to 182.3, *I*_2_ = 83%, *p =* 0.005) (Fig 2). In keeping with the overall lower birth weight in the metformin-exposed group, macrosomia was lower by 40% compared to the insulin-exposed group (OR 0.59, 95% CI 0.46 to 0.77, *I*_2_ = 0%, *p <* 0.001), although this result may be influenced by publication bias (Fig 3). LGA was also lower in metformin-exposed neonates compared to insulin-exposed neonates (OR 0.78, 95% CI 0.62 to 0.99, *I*_2_ = 7%, *p =* 0.04) (Fig 4). However, the difference in LGA between metformin- and insulin-exposed neonates was no longer significant when leave-one-out analysis was performed (S3 Fig). There was no difference in the risk of being born SGA (OR 0.99, 95% CI 0.68 to 1.46, *I*_2_ = 0%) (Fig 5) or in neonatal height (mean height difference 0.03 cm, 95% CI −0.71 to 0.77, *I*_2_ = 78%) between the treatment groups (Fig 6).

**Fig 7:**
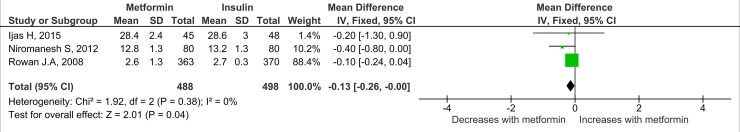
Neonatal ponderal index. Expressed as mean difference (fixed effects model) and 95% CI. IV, mean difference.

**Fig 8:**
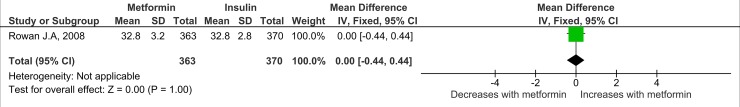
Neonatal abdominal circumference. Expressed as mean difference (fixed effects model) and 95% CI. IV, mean difference.

**Fig 9:**
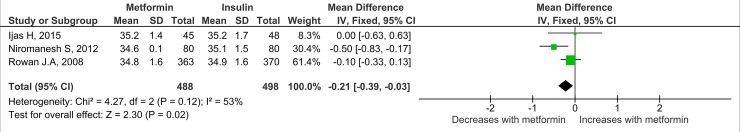
Neonatal head circumference. Expressed as mean difference (fixed effects model) and 95% CI. IV, mean difference.

**Fig 10:**
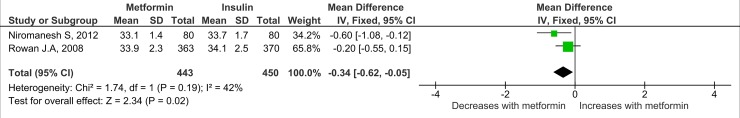
Neonatal chest circumference. Expressed as mean difference (fixed effects model) and 95% CI. IV, mean difference.

Three studies reported neonatal ponderal index [15,16,29] (*n =* 986 neonates). Neonatal ponderal index was significantly lower in the metformin-exposed compared with the insulin-exposed group (−0.13, 95% CI −0.26 to −0.00; *I*_2_ = 0%, *p =* 0.04) (Fig 7). Only 1 study [16] (*n =* 733 neonates) reported neonatal abdominal circumference, which was not significantly different between groups (Fig 8). However, 3 studies reported neonatal head circumference [15,16,29] (*n =* 986 neonates), which was significantly lower in the metformin-exposed group (−0.21 cm, 95% CI −0.39 to −0.03, *I*_2_ = 53%, *p =* 0.02) (Fig 9) compared to the insulin-exposed group, as was chest circumference, which was reported in 2 studies [16,29] (*n =* 893 neonates) (−0.34 cm, 95% CI −0.62 to −0.05, *I*_2_ = 42%, *p =* 0.02) (Fig 10).

### Infant growth (18 months–2 years) and childhood growth (5–9 years)

**Fig 11:**
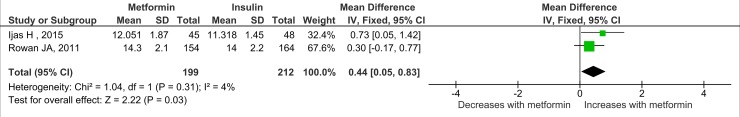
Infant weight. Expressed as mean differences (fixed effects model) and 95% CI. IV, mean difference.

**Fig 12:**
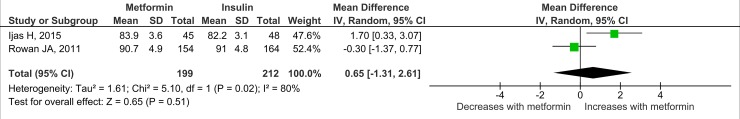
Infant height. Expressed as mean differences (random effects model) and 95% CI. IV, mean difference.

Two studies [16,36] (*n =* 411 infants) reported weight during infancy (between 18 months and 2 years of age). Infants of mothers treated with metformin during pregnancy were significantly heavier than infants of mothers treated with insulin during pregnancy (mean difference 440 g, 95% CI 50 to 830, *I*_2_ = 4%, *p =* 0.03) (Fig 11). However, infant height was not significantly different between treatment groups (0.65 cm, 95% CI −1.31 to 2.61, *I*_2_ = 80%) (Fig 12).

**Fig 13:**
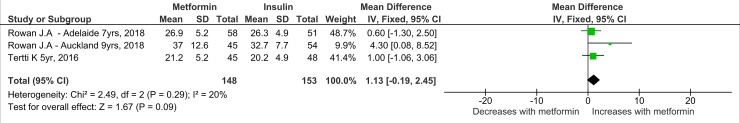
Childhood weight. Expressed as mean differences (fixed effects model) and 95% CI. IV, mean difference.

**Fig 14:**
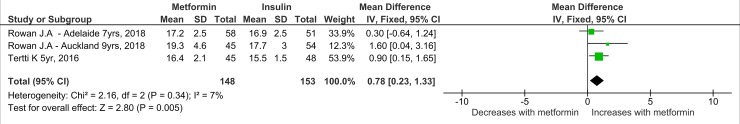
Childhood BMI. Expressed as mean differences (fixed effects model) and 95% CI. IV, mean difference.

**Fig 15:**
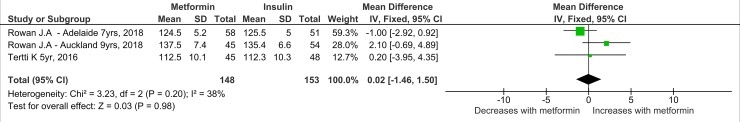
Childhood height. Expressed as mean differences (fixed effects model) and 95% CI. IV, mean difference.

Two studies [38,41] reported weight during mid-childhood (*n =* 301 children). One of these studies [41], reported outcomes from 2 different sites. Children of mothers treated with metformin during pregnancy tended to be heavier than children of mothers treated with insulin during pregnancy, but this difference did not reach statistical significance (mean difference 1.13 kg, 95% CI −0.19 to 2.45, *I*_2_ = 20%, *p =* 0.09) (Fig 13). However, childhood BMI, reported in 2 studies [38,41] (*n =* 301 children), was significantly higher in the metformin-exposed group than in the insulin-exposed group (mean difference 0.78 kg/m^2^, 95% CI 0.23 to 1.33, *I*_2_ = 7%, *p =* 0.005) (Fig 14). As with height at birth and in infancy, there was no difference in height in childhood between the treatment groups (Fig 15). A follow-up study (*n =* 208 children) [41] from different sites of a single randomised controlled trial [16] examined anthropometry and body composition in mid-childhood. None of the measured growth parameters (head, chest, and waist circumference, and weight:height ratio) was significantly different between the 2 treatment groups (S5 Fig). However, this study demonstrated [41] higher abdominal (*p =* 0.02) and visceral (*p =* 0.03) fat volumes in metformin-exposed children compared to the children whose mothers were treated with insulin during pregnancy (Table 1). There were no significant differences between the groups in terms of total fat mass or abdominal fat mass assessed by dual X-ray absorptiometry scanning in mid-childhood (Table 1).

**Table 1:** Childhood adiposity indices.

Adiposity index	Study details	Mean difference (fixed)	95% CI	Significance	Heterogeneity test
Total fat mass (DEXA)	Adelaide (*n =* 61) Auckland (*n =* 98)	0.20	−0.11, 0.51	*p =* 0.25	*p =* 0.15, *I*_2_ = 52%
Abdominal fat mass (DEXA)	Adelaide (*n =* 61) Auckland (*n =* 98)	79.80	−59.32, 218.92	*p =* 0.26	*p =* 0.11, *I*_2_ = 60%
Abdominal fat volume (MRI)	Adelaide (*n =* 12) Auckland (*n =* 92)	0.44	0.06, 0.82	***p =* 0.02**	*p =* 0.84, *I*_2_ = 0%
Abdominal subcutaneous fat volume (MRI)	Adelaide (*n =* 12) Auckland (*n =* 92)	0.29	−0.07, 0.65	*p* = 0.11	*p =* 0.95, *I*_2_ = 0%
Visceral fat volume (MRI)	Adelaide (*n =* 12) Auckland (*n =* 92)	0.41	0.05, 0.77	***p =* 0.03**	*p =* 0.85, *I*_2_ = 0%
Thigh fat mass (DEXA)	Adelaide (*n =* 61) Auckland (*n =* 98)	90.77	−148.68, 330.23	*p =* 0.46	*p =* 0.46, *I*_2_ = 61%
Arm fat mass (DEXA)	Adelaide (*n =* 61) Auckland (*n =* 98)	102.57	−73.34, 278.47	*p =* 0.25	*p =* 0.09, *I*_2_ = 65%
Bicep skinfold thickness	Adelaide (*n =* 109) Auckland (*n =* 98)	0.53	−0.60, 1.66	*p =* 0.35	*p =* 0.21, *I*_2_ = 36%
Tricep skinfold thickness	Adelaide (*n =* 109) Auckland (*n =* 98)	0.64	−0.76, 2.04	*p =* 0.83	*p =* 0.37, *I*_2_ = 70%
Subscapular skinfold thickness	Adelaide (*n =* 109) Auckland (*n =* 98)	1.05	−0.70, 2.79	*p =* 0.24	*p =* 0.32, *I*_2_ = 0%

All outcomes expressed as mean difference, with the exception of abdominal, abdominal subcutaneous, and visceral fat volumes, which are expressed as standard mean difference. All outcomes used fixed model effects and 95% CI. Significant *p-*values are in bold. Units: fat mass, grams; fat volume, cm^3^; skinfold thickness, millimetres. All data from Rowan et al. [41]. DEXA, dual X-ray absorptiometry.

## Discussion

In this study of randomised evidence, we found that neonates exposed to metformin in utero weighed less at birth than neonates whose mothers were exposed to insulin, in the context of treatment for GDM. The risk of macrosomia is substantially lower, by 40%, when GDM is treated with metformin compared to insulin, without a concomitant increase in the risk of being born SGA. The limited number of studies that have addressed neonatal anthropometry suggest that metformin-exposed neonates have lower lean mass compared to neonates whose mothers were treated with insulin, in terms of lower ponderal index, head circumference, and chest circumference with no change in abdominal circumference.

Despite being born at lower average birth weights, by the age of 2 years, metformin-exposed infants were heavier than infants whose mothers were treated with insulin [36,40]. In mid-childhood (5–9 years), the absolute weight difference between groups did not reach statistical significance [38,40], but children exposed to metformin in utero had higher BMI (by 0.78 kg/m^2^) than those whose mothers were treated with insulin [38,40]. Very limited data were available on body composition in infancy or childhood, but those available showed no significant differences on any specific anthropometric parameters [41]. Follow-up studies (from 2 different sites) based on a single original trial [16] also included detailed assessments of adiposity in childhood [41]. These suggested that the higher BMI observed in children exposed to metformin in utero compared to those exposed to insulin may be the result of greater abdominal adiposity (as evidenced by higher visceral and abdominal fat volumes), which is known to be strongly associated with cardio-metabolic disease in later life [47].

### Strengths

A major strength of our meta-analysis is our ability to provide a complete overview of the effect of intrauterine metformin exposure on perinatal growth and body composition via serial meta-analyses in discrete developmental windows [48–55]. Our study design maximally leverages available data to provide the most complete possible analysis of growth patterns from intrauterine development to mid-childhood using available data. Our findings highlight the lack of published serial fetal growth data from any randomised trial that met the inclusion criteria for this study.

Previous studies have examined various outcomes of intrauterine metformin exposure in mixed populations with a variety of indications [56]. A further strength of our study was that in order to standardise expected growth trajectories as far as possible, we limited our analysis to studies of women with confirmed GDM. GDM is associated with accelerated fetal growth trajectories [57], and thus any impact of metformin exposure on perinatal growth trajectories is likely to be magnified and more readily detectable in GDM-affected populations. Moreover, GDM is the most common clinical indication for use of metformin during pregnancy, which is endorsed in many national guidelines, including in the UK [58,59] and New Zealand [60], and by societies including the International Federation of Gynecology and Obstetrics [61] and SMFM [19], making our study findings more directly applicable to clinical practice.

A further strength of our meta-analysis is that our findings appear robust across different global populations, including both high and low-middle development index settings. Furthermore, other large well-conducted studies in which women were randomised to metformin treatment during pregnancy for other indications reinforce our finding of altered postnatal body composition following in utero metformin exposure. Vanky and colleagues studied children of women treated with metformin for polycystic ovary syndrome. Their study [62] included 160 children from 2 sites in Norway who were followed up at the age of 4 years. They showed that those who were exposed to metformin in utero had increased weight *z*-score (difference in means 0.38, 95% CI 0.07 to 0.69), increased risk of overweight/obesity (OR 2.17, 95% CI 1.04 to 4.61), and increased BMI (difference in means 0.45 kg/m^2^, 95% CI 0.11 to 0.78) compared to those who were exposed to placebo in utero. When these children (*n =* 144) were followed up at 5–10 years of age [63], children whose mothers were treated with metformin maintained the increased BMI observed at 4 years of age (difference in means 0.41 kg/m^2^, 95% CI 0.03 to 0.78). Moreover, data from a randomised trial of 449 overweight or obese normoglycaemic women randomised to metformin or placebo from 16 weeks of gestation (EMPOWaR), showed that neonates exposed to metformin compared to placebo in utero were thinner at birth (lower neonatal ponderal index), suggesting very early differences in body composition [64,65]. Our confidence in our finding of a higher tendency towards lower lean mass and greater adiposity in metformin-exposed compared to insulin-exposed children is increased by these studies.

### Limitations

The ability to draw definitive conclusions from our meta-analysis is limited by both the quantity and quality of the studies available. In particular, longitudinal follow-up data into mid-childhood from trials of GDM treatment are sparse in comparison to earlier time points (110–301 children from 3 studies). Where follow-up data are available, the original studies may be subject to recall bias and power issues with respect to childhood outcomes. The majority of clinical trials in this area are powered only for primary outcomes at the time of birth. Our findings highlight a need for further longitudinal studies of growth and body composition following intrauterine metformin exposure.

The overall lower incidence of macrosomia and the average lower birth weight associated with metformin compared to insulin treatment in trial contexts has been interpreted as evidence of efficacy and beneficial impact on fetal growth. However, our meta-analysis demonstrates a lack of data on which to base conclusions regarding fetal growth trajectories and neonatal body compositions associated with lower birth weight. In the absence of such data, it is challenging to determine whether lower birth weight represents lower lean mass (as possibly indicated by a lower ponderal index) or lower fetal adiposity (in keeping with maintenance of normoglycaemia in GDM). This uncertainty underlines the necessity for studies examining the consequences of intrauterine metformin exposure for fetal growth and postnatal catch-up growth, which is associated with cardiovascular and metabolic disease risk in later life [48–55].

Regarding study quality, there was considerable risk of bias associated with a number of studies, particularly with regard to poorly described or quasi-randomisation procedures [24–26,31,42,44,45]. In all trials, women who were randomised to, but not successfully treated with, metformin (defined as failure to maintain plasma glucose within the defined range) were subsequently offered insulin treatment. Several trials then excluded women who required both metformin and insulin treatment from the final analyses [24–26,28,30,34], rather than continuing to analyse their data on an intention-to-treat basis. Women who were not adequately treated with metformin ranged from 14% to 46% of the total randomised to metformin. Hence, the exclusion of these women may represent the introduction of serious bias into the trial results. In particular, the women who were not successfully treated with metformin alone may have had more adverse outcomes overall, and so their exclusion may skew the effect estimates compared to studies where intention-to-treat analysis was performed. In order to assess whether bias introduced through this route impacted the meta-analysis results overall, we conducted a sensitivity analysis excluding studies in which analysis was not performed on an intention-to-treat basis. As exclusion of these studies did not substantively alter the results of any reported outcome, we have included studies that were not analysed on an intention-to-treat basis in our study. An additional limitation is the inter-study heterogeneity in the diagnostic criteria used for GDM, reflecting the geographical and date spread of the included studies. In total, 8 different diagnostic criteria were used. Furthermore, in some studies the diagnostic criteria were changed during recruitment [17,37,38,66]. Diagnosing GDM at lower glucose intolerance thresholds may decrease the effect size of the observed response to treatment. However, our sensitivity analysis with respect to diagnostic criteria indicates that this did not materially alter the overall conclusions.

### Interpretation

We show that metformin-exposed babies are born smaller than their insulin-exposed counterparts, with lower ponderal index, but then undergo accelerated postnatal growth such that by infancy they have higher weight and by childhood they have higher BMI. This is a concerning finding, as previous evidence from a variety of contexts suggests that such patterns of low birth weight and postnatal catch-up growth can be associated with adverse long-term cardio-metabolic outcomes [48–55].

Metformin readily crosses the placenta via organic cation transporters and may reach concentrations in the fetus approaching those in the mother [22,23]. Organic cation transporters are present in both placental and fetal tissues in the second and third trimester, which is the period during which treatment for GDM takes place [67]. Metformin has a variety of intracellular effects, including inhibition of mitochondrial respiration [68] and effects on the mTOR pathway, by both AMPK-dependent [69] and AMPK-independent [70] mechanisms. mTOR is important for nutrient sensing, and it is known that metformin can act upon mTOR and cause nutrient restriction of glucose and amino acids to the fetus and placenta [71]. The growth pattern seen in infants exposed to metformin compared to insulin for treatment of GDM (low birth weight followed by accelerated post-natal catch-up growth) closely resembles that of the nutritionally deprived fetus. Such growth patterns have been reported in several studies to be associated with adverse cardio-metabolic consequences in later life including obesity, type 2 diabetes, and cardiovascular disease [48–55]. Data on the very long-term consequences of fetal metformin exposure are currently lacking, as are data on intrauterine growth trajectories, detailed perinatal phenotyping, and placental effects of metformin exposure. These are important future research priorities. The impact of other oral glucose-lowering agents used in GDM treatment, for example glyburide, which is used in certain countries such as the US [9], was not addressed in the current analysis. The impact of such treatment on fetal, infant, and long-term growth is therefore another important research priority.

Our observations are timely because the incidence of GDM is increasing rapidly in many populations, with 1 in 7 of all babies being born to GDM-affected mothers in 2017 globally [72]. Importantly, the incidence of GDM is also rising in populations in which treating GDM with insulin is unlikely to be feasible for large numbers of women [73]. Treating GDM with metformin is becoming more common and is being introduced into a growing number of countries (reviewed in [74]); thus, it is a research priority to ensure that such treatment is not detrimental to long-term health [75].

It has been demonstrated in many studies that the patterns of growth in in utero and early postnatal periods are associated with long-term metabolic consequences, including obesity, cardiovascular disease, and impaired glucose tolerance [48–55,76,77]. The current findings demonstrate that metformin treatment of GDM alters the postnatal growth trajectory compared to insulin treatment. Children exposed to metformin compared to insulin in utero were born at significantly lower birth weights, but were significantly heavier in infancy, with higher BMI by mid-childhood. These data warrant further detailed investigation of the implications of treating GDM with metformin, a practice that is currently endorsed in several settings worldwide.

## Supporting information

S1 PRISMA Checklist (DOC)

S1 Fig Leave-one-criterion-out sensitivity analysis for birth weight. (A) Minus American Diabetes Association (ADA), (B) minus Australasian Diabete**s** in Pregnancy Society (ADIPS), (C) minus American College of Obstetricians and Gynecologists (ACOG), (D) minus Carpenter–Coustan (CC), (E) minus Finnish national criteria, (F) minus International Association of Diabetes and Pregnancy Study Groups (IADPSG), (G) minus National Diabetes Data Group (NDDG) (H) minus World Health Organization (WHO), and (I) minus studies without GDM criteria details.
(PPTX)

S2 Fig Subgroup analysis exploring whether removal of biased studies affects outcomes. (A) Birth weight, (B) macrosomia, (C) LGA, and (D) SGA. All outcomes expressed as OR (95% CI), with the exception of birth weight, which is expressed as mean difference (95% CI).
(PPTX)

S3 Fig Leave-one-out sensitivity analysis. (A) Birth weight, (B) macrosomia, (C) LGA, and (D) SGA. All outcomes expressed as OR (95% CI).
(PPTX)

S4 Fig Funnel plots to assess publication bias. All outcomes plotted.
(PPTX)

S5 Fig Childhood (7–9 years) anthropometry. (A) Childhood head circumference, (B) childhood chest circumference, (C) childhood waist circumference, and (D) childhood waist:hip ratio. All outcomes expressed as mean differences (95% CI).
(PPTX)

S1 Table Inclusion/exclusion criteria. (PPTX)

S2 Table Risk-of-bias assessment. (A) Random sequence generation (selection bias), (B) allocation concealment (selection bias), (C) blinding participants and personnel (performance bias), (D) blinding of outcome assessment (detection bias), (E) incomplete oucome data (attrition bias), (F) selection bias (reporting bias), and (G) other bias.
(PPTX)

S3 Table Study characteristics. (XLSX)

S1 Text PROSPERO protocol. CRD42018117503.
(PPTX)

S2 Text Database search strategies. (A) PubMed, (B) Ovid Embase, (C) Medline, (D) Web of Science, (E) Cochrane Library, and (F) ClinTrials.gov.
(PPTX)

## Abbreviations

GDM: gestational diabetes mellitus
LGA: large for gestational age
OR: odds ratio
SGA: small for gestational age
SMFM: Society for Maternal–Fetal Medicine
