# Supplementary material for: Neonatal, infant, and childhood growth following metformin versus insulin treatment for gestational diabetes: A systematic review and meta-analysis
Source: PLoS Med. 2019 Aug 6;16(8):e1002848. doi: 10.1371/journal.pmed.1002848 (PMC6684046; doi:10.1371/journal.pmed.1002848)
Supplement: S1 PRISMA Checklist — (DOC) [file pmed.1002848.s001.doc]

| **Section/topic** | **#** | **Checklist item** | **Reported on page #** |
| --- | --- | --- | --- |
| **TITLE** | | |  |
| Title | 1 | Identify the report as a systematic review, meta-analysis, or both. “Fetal, infant, and childhood growth following metformin versus insulin treatment for gestational diabetes: a systematic review and meta-analysis”. | Title, paragraph 1 |
| **ABSTRACT** | | |  |
| Structured summary | 2 | Provide a structured summary including, as applicable: background; objectives; data sources; study eligibility criteria, participants, and interventions; study appraisal and synthesis methods; results; limitations; conclusions and implications of key findings; systematic review registration number. “**Background:** Metformin is increasingly offered as an acceptable and economic alternative to insulin for treatment of gestational diabetes (GDM) in many countries. However, the impact of maternal metformin treatment on the trajectory of fetal, infant, and childhood growth is not known. **Methods and findings:** PubMed, OVID EMBASE, Medline, Web of Science, clinicaltrials.gov and the Cochrane database were systematically searched (from database inception to 26^th^ February 2019)...” | Abstract, paragraphs 2-6. |
| **INTRODUCTION** | | |  |
| Rationale | 3 | Describe the rationale for the review in the context of what is already known. “Gestational diabetes mellitus (GDM) currently affects between 3-25% of pregnancies worldwide (1), constituting a significant global health-care burden. The continuing increase in the global incidence of GDM may relate to new screening approaches, decreasing threshold values for diagnosis, or to increases in population risk factors, particularly obesity (2, 3). GDM poses significant risks…” | Introduction, paragraph 7. |
| Objectives | 4 | Provide an explicit statement of questions being addressed with reference to participants, interventions, comparisons, outcomes, and study design (PICOS). “The aim of our study was therefore to determine whether metformin compared to insulin treatment for GDM alters perinatal growth trajectories or body composition from the time of fetal exposure through to mid-childhood. Addressing this issue is particularly urgent as the number of pregnancies exposed to metformin increases worldwide (8). | Introduction, paragraph 10. |
| **METHODS** | | |  |
| Protocol and registration | 5 | Indicate if a review protocol exists, if and where it can be accessed (e.g., Web address), and, if available, provide registration information including registration number. “This systematic review and meta-analysis was conducted in accordance with the Preferred Reporting Items for Systematic Reviews and Meta-Analyses (PRISMA) guidelines (31). The systematic review protocol was registered in PROSPERO (CRD42018117503) (Supplementary Figure 1). Ethical approval was not required”. | Materials and methods, paragraph 11. Supplementary Figure . |
| Eligibility criteria | 6 | Specify study characteristics (e.g., PICOS, length of follow-up) and report characteristics (e.g., years considered, language, publication status) used as criteria for eligibility, giving rationale. “Studies that randomised women with GDM to metformin *versus* insulin therapy were included. Studies were excluded if they compared metformin exposure to other oral glucose-lowering agents (e.g. glyburide) or if interventions were given prior to pregnancy. GDM was screened for and diagnosed according to local criteria in each study, and we did not apply exclusions with respect to this. Studies…” | Materials and methods, paragraph 13. |
| Information sources | 7 | Describe all information sources (e.g., databases with dates of coverage, contact with study authors to identify additional studies) in the search and date last searched. “Systematic literature searches using pre-specified terms (Supplementary Figure 2) were performed on PubMed (June 1997 to 26^th^ February 2019), Ovid EMBASE (1974 to 26^th^ February 2019), Ovid Medline (1946 to 26^th^ February 2019), Cochrane library (database inception to 26^th^ February 2019), Clinicaltrials.gov (database inception to 26th February 2019), and Web of Science (1900 to 26^th^ February 2019). No filters were applied to any of the searches. No language or location restrictions were applied.” | Materials and methods, paragraph 12. |
| Search | 8 | Present full electronic search strategy for at least one database, including any limits used, such that it could be repeated.  “(A) Search criteria for PubMed: ("metformin"[MeSH Terms] OR "metformin"[All Fields]) AND ("diabetes, gestational"[MeSH Terms] OR ("diabetes"[All Fields] AND "gestational"[All Fields]) OR "gestational diabetes"[All Fields] OR ("gestational"[All Fields] AND "diabetes"[All Fields])) AND ("pregnancy in diabetics"[MeSH Terms] OR ("pregnancy"[All Fields] AND "diabetics"[All Fields]) OR "pregnancy in diabetics"[All Fields] OR ("diabetes"[All Fields] AND "pregnancy"[All Fields]) OR "diabetes in pregnancy"[All Fields] OR "diabetes, gestational"[MeSH Terms] OR ("diabetes"[All Fields] AND "gestational"[All Fields]) OR "gestational diabetes"[All Fields] OR ("diabetes"[All Fields] AND "pregnancy"[All Fields])) … | Supplementary Figure |
| Study selection | 9 | State the process for selecting studies (i.e., screening, eligibility, included in systematic review, and, if applicable, included in the meta-analysis). “Two reviewers (JLA and CEA) independently assessed each study using pre-determined inclusion/exclusion criteria (Supplementary Table 1). A third reviewer (SEO) was available to resolve cases where eligibility was unclear. An initial screen of titles and abstracts was performed, followed by a detailed full paper screen. The results from each step of the review process are documented in a PRISMA flow diagram (Figure 1)”. | Materials and methods. Paragraph 14. Supp Table 1 & Figure 1. |
| Data collection process | 10 | Describe method of data extraction from reports (e.g., piloted forms, independently, in duplicate) and any processes for obtaining and confirming data from investigators. “Data extraction from eligible studies was conducted independently by two authors (JLA and CEA)”. “Where insufficient information for assessment was available, authors were contacted for further information.” | Materials and methods. Paragraph 15 and  Paragraph 13. |
| Data items | 11 | List and define all variables for which data were sought (e.g., PICOS, funding sources) and any assumptions and simplifications made. “Outcome measures were: fetal growth parameters (head circumference, abdominal circumference, femur length, biparietal diameter, estimated fetal weight calculated by any formula; mm or grams), birth-weight (grams or kilograms), SGA (birth-weight <10^th^ centile for gestational age; n values and %), LGA (birth-weight >90^th^ centile for gestational age; n values and %), macrosomia…” | Materials and methods. Paragraph 15. |
| Risk of bias in individual studies | 12 | Describe methods used for assessing risk of bias of individual studies (including specification of whether this was done at the study or outcome level), and how this information is to be used in any data synthesis. “Each study was independently assessed by two authors (JLA and CEA) for quality and validity using the Cochrane Collaboration tool for assessing Risk of Bias. Seven risk of bias domains were assessed for each study and each domain was given a rating of low risk, unknown risk or high risk of bias (Supplementary Figure 3). All risk of bias analysis was conducted at the study level”. | Materials and methods. Paragraph 16. |
| Summary measures | 13 | State the principal summary measures (e.g., risk ratio, difference in means). “The principle summary measures utilised in this systematic review were unadjusted odds ratios (for dichotomous data) or differences in means (for continuous data)”. | Materials and methods. Paragraph 17. |
| Synthesis of results | 14 | Describe the methods of handling data and combining results of studies, if done, including measures of consistency (e.g., I^2^) for each meta-analysis. “Meta-analysis was performed using Review Manager ((RevMan) [Computer program] Version 5.3, Copenhagen: The Nordic Cochrane Centre, the Cochrane Collaboration, 2014) and the ‘*metafor*’ package in R (version 3.5.1 [R core]). Funnel plots were…” | Materials and methods. Paragraph 17. |

| Risk of bias across studies | 15 | Specify any assessment of risk of bias that may affect the cumulative evidence (e.g., publication bias, selective reporting within studies). “Funnel plots were constructed to assess publication bias and outcome measures with 5 or more studies included were also subjected to Egger’s test.”. | Materials and methods. Paragraph 17. |
| --- | --- | --- | --- |
| Additional analyses | 16 | Describe methods of additional analyses (e.g., sensitivity or subgroup analyses, meta-regression), if done, indicating which were pre-specified. “Heterogeneity between studies was assessed using the I-squared statistic, and any outcomes showing significant inter-study heterogeneity were analysed using a random-effects model. Sensitivity analyses were performed using leave-one-out testing, and by conducting meta-analyses using only the sub-set of studies assessed as having a low-moderate risk of bias”. | Materials and methods. Paragraph 17. |
| **RESULTS** | | |  |
| Study selection | 17 | Give numbers of studies screened, assessed for eligibility, and included in the review, with reasons for exclusions at each stage, ideally with a flow diagram. “Electronic searching of the specified data-bases yielded a total of 2559 studies and 1 further study was found via hand-searching. After removal of duplicates and title/abstract screening, 121 trials were screened for full text assessment, applying the full set of eligibility criteria (Figure 1). After full-text evaluation, a total of 28 studies remained eligible for inclusion, representing 3976 pregnancies (Figure 1)”. | Results. Paragraph 18 and Figure 1. |
| Study characteristics | 18 | For each study, present characteristics for which data were extracted (e.g., study size, PICOS, follow-up period) and provide the citations. “The included studies varied in terms of quality and design (Supplementary Table 2). Outcomes measured varied between studies, with birth-weight the most commonly reported outcome (17 studies). There was considerable clinical heterogeneity in the dose of metformin used (ranging from 500mg to 3000mg daily) both within and between studies. There was a range of geographical settings including Europe (15, 17, 33-35), USA (36), Australia/New Zealand (16, 37, 38), south Asia (29, 39) and the Middle East (22-28, 32, 40-43)”. | Results, paragraph 19 and Supplementary Table 2 |
| Risk of bias within studies | 19 | Present data on risk of bias of each study and, if available, any outcome level assessment (see item 12).  “The risk of bias was moderate-to-low in the majority of included studies. However, six studies did not analyse data on an intention-to-treat basis (i.e. trial participants who did not achieve adequate glycaemic control with metformin were removed from the study (22-24, 26, 28, 32), leading to a high risk of bias). A further study had significant imbalance in the baseline characteristics of participants, potentially due to failure of randomisation (39). We performed sub-group meta-analyses, excluding the studies assessed as having a high risk of bias...” | Results, paragraph 20. |
| Results of individual studies | 20 | For all outcomes considered (benefits or harms), present, for each study: (a) simple summary data for each intervention group (b) effect estimates and confidence intervals, ideally with a forest plot. “19 studies (n=3,723 neonates) reported neonatal growth parameters. Birth-weights of neonates born to mothers treated with metformin were significantly lower than neonates whose mothers were treated with insulin during pregnancy. On average, metformin-exposed neonates were 107.7g smaller than those whose mothers were randomised to insulin (95% CI: 32.7 to 182.3g; I_2_=83%, p=0.005) (Figure 2 A)…” | Results, paragraphs 22-26. Figures 2- 4. Supplementary Figure 7, Table 1. |
| Synthesis of results | 21 | Present results of each meta-analysis done, including confidence intervals and measures of consistency. “19 studies (n=3,723 neonates) reported neonatal growth parameters. Birth-weights of neonates born to mothers treated with metformin were significantly lower than those treated with insulin during pregnancy. On average, metformin-exposed neonates were 107.7g smaller than those whose mothers were randomised to insulin (95% CI: 32.7 to 182.3g; I_2_=83%, p=0.005) (Figure 2 A)...” | Results, paragraphs 22 - 26. Figures 2- 4. Supplementary Figure 7, Table 1. |
| Risk of bias across studies | 22 | Present results of any assessment of risk of bias across studies (see Item 15). “Funnel plots for all outcomes were assessed visually (Supplementary Figure 6). There were no obvious asymmetries in the plots for any study outcomes, with the exception of macrosomia. Egger’s test (p<0.05) confirmed the likelihood of publication bias with respect to macrosomia, but confirmed that there was no reason to believe that significant publication bias affected any of the other outcomes studied”. | Results, paragraph 20. |
| Additional analysis | 23 | Give results of additional analyses, if done (e.g., sensitivity or subgroup analyses, meta-regression [see Item 16]). “We performed sub-group meta-analyses, excluding the studies assessed as having a high risk of bias. Sub-group analysis of low-moderate risk studies only, showed…” that removal of the high-risk studies did not materially alter the outcome of the meta-analysis for any of the outcomes assessed, therefore all studies were included (Supplementary Figure 4)”. | Results, paragraph 20. Supplementary Figures 5 and 6. |
| **DISCUSSION** | | |  |
| Summary of evidence | 24 | Summarize the main findings including the strength of evidence for each main outcome; consider their relevance to key groups (e.g., healthcare providers, users, and policy makers). “In this study of randomized evidence, we found that neonates exposed to metformin *in utero* weighed less at birth than those neonates whose mothers were exposed to insulin in the context of treatment for GDM. The risk of macrosomia is substantially reduced by 40% when GDM is treated with metformin compared to insulin without a concomitant increase in the risk of being born small-for-gestational age. The limited number of studies that have addressed neonatal anthropometry suggest that metformin-exposed neonates have reduced lean mass compared to those neonates whose mothers were treated with insulin, in terms of reduced ponderal index, head circumference, and chest circumference with no change in abdominal circumference. Despite being born at lower average birth-weights…” “Growth trajectories *in utero* and in the early postnatal period are associated with long-term metabolic consequences, including obesity and impaired glucose tolerance (45-52). Metformin treatment of GDM alters post-natal growth trajectory compared to insulin treatment. Children exposed to metformin *in utero* were born at significantly lower birth-weights, but were significantly heavier in infancy, with higher BMI by mid-childhood. These data warrant further detailed investigation of the implications for treating GDM with metformin, a practice that is currently endorsed in several settings worldwide”. | Discussion, paragraphs 27-28.  Discussion, paragraph 38. |
| Limitations | 25 | Discuss limitations at study and outcome level (e.g., risk of bias), and at review-level (e.g., incomplete retrieval of identified research, reporting bias). “The certainty associated with the findings of our meta-analysis is limited by both the quantity and quality of the studies available. In particular, longitudinal follow-up data into childhood from trials of GDM treatment are sparse in comparison to earlier time-points (between 110-301 children). Where follow-up data are available, the original studies may be subject to recall bias and power issues with respect to childhood outcomes. The majority of clinical trials in this area are powered only for primary outcomes at the time of birth. Our findings highlight an urgent need for further longitudinal studies of growth and body composition following intrauterine metformin-exposure…” | Discussion, paragraphs 32-34. |
| Conclusions | 26 | Provide a general interpretation of the results in the context of other evidence, and implications for future research. “Growth trajectories *in utero* and in the early postnatal period are associated with long-term metabolic consequences, including obesity and impaired glucose tolerance (45-52). Metformin treatment of GDM alters post-natal growth trajectory compared to insulin treatment. Children exposed to metformin *in utero* were born at significantly lower birth- weights, but were significantly heavier in infancy, with higher BMI by mid-childhood. These data warrant further detailed investigation of the implications for treating GDM with metformin, a practice that is currently endorsed in several settings worldwide. | Discussion, paragraph 38.  Paragraphs 35-37. |
| **FUNDING** | | |  |
| Funding | 27 | Describe sources of funding for the systematic review and other support (e.g., supply of data); role of funders for the systematic review. Funding sources not required in manuscript. This is provided in the financial disclosure section of the manuscript submission system, as requested by the Journal. | N/A |

*From:*  Moher D, Liberati A, Tetzlaff J, Altman DG, The PRISMA Group (2009). Preferred Reporting Items for Systematic Reviews and Meta-Analyses: The PRISMA Statement. PLoS Med 6(7): e1000097. doi:10.1371/journal.pmed1000097

For more information, visit: **www.prisma-statement.org**.

Page 4 of 4

**Supporting Information S1: PRISMA 2009 Checklist.**
