# Supplementary material for: Neonatal, infant, and childhood growth following metformin versus insulin treatment for gestational diabetes: A systematic review and meta-analysis
Source: PLoS Med. 2019 Aug 6;16(8):e1002848. doi: 10.1371/journal.pmed.1002848 (PMC6684046; doi:10.1371/journal.pmed.1002848)
Supplement: S1 Fig — (A) Minus American Diabetes Association (ADA), (B) minus Australasian Diabetes in Pregnancy Society (ADIPS), (C) minus American College of Obstetricians and Gynecologists (ACOG), (D) minus Carpenter–Coustan (CC), (E) minus Finnish national criteria, (F) minus International Association of Diabetes and Pregnancy Study Groups (IADPSG), (G) minus National Diabetes Data Group (NDDG) (H) minus World Health Organization (WHO), and (I) minus studies without GDM criteria details. (PPTX) [file pmed.1002848.s002.pptx]

## Slide 1
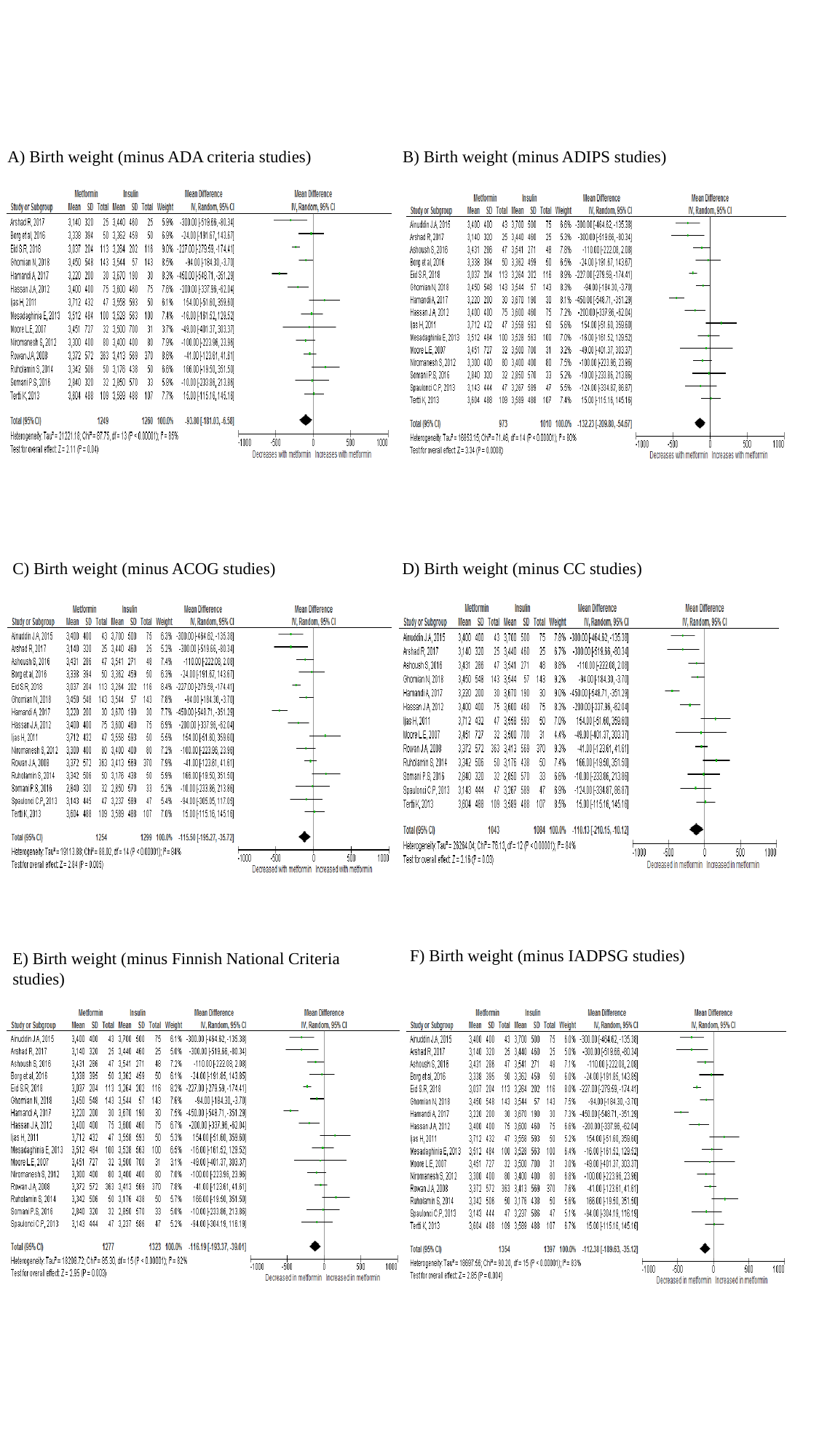

A) Birth weight (minus ADA criteria studies)
B) Birth weight (minus ADIPS studies)
C) Birth weight (minus ACOG studies)
D) Birth weight (minus CC studies)
F) Birth weight (minus IADPSG studies)
E) Birth weight (minus Finnish National Criteria studies)

## Slide 2
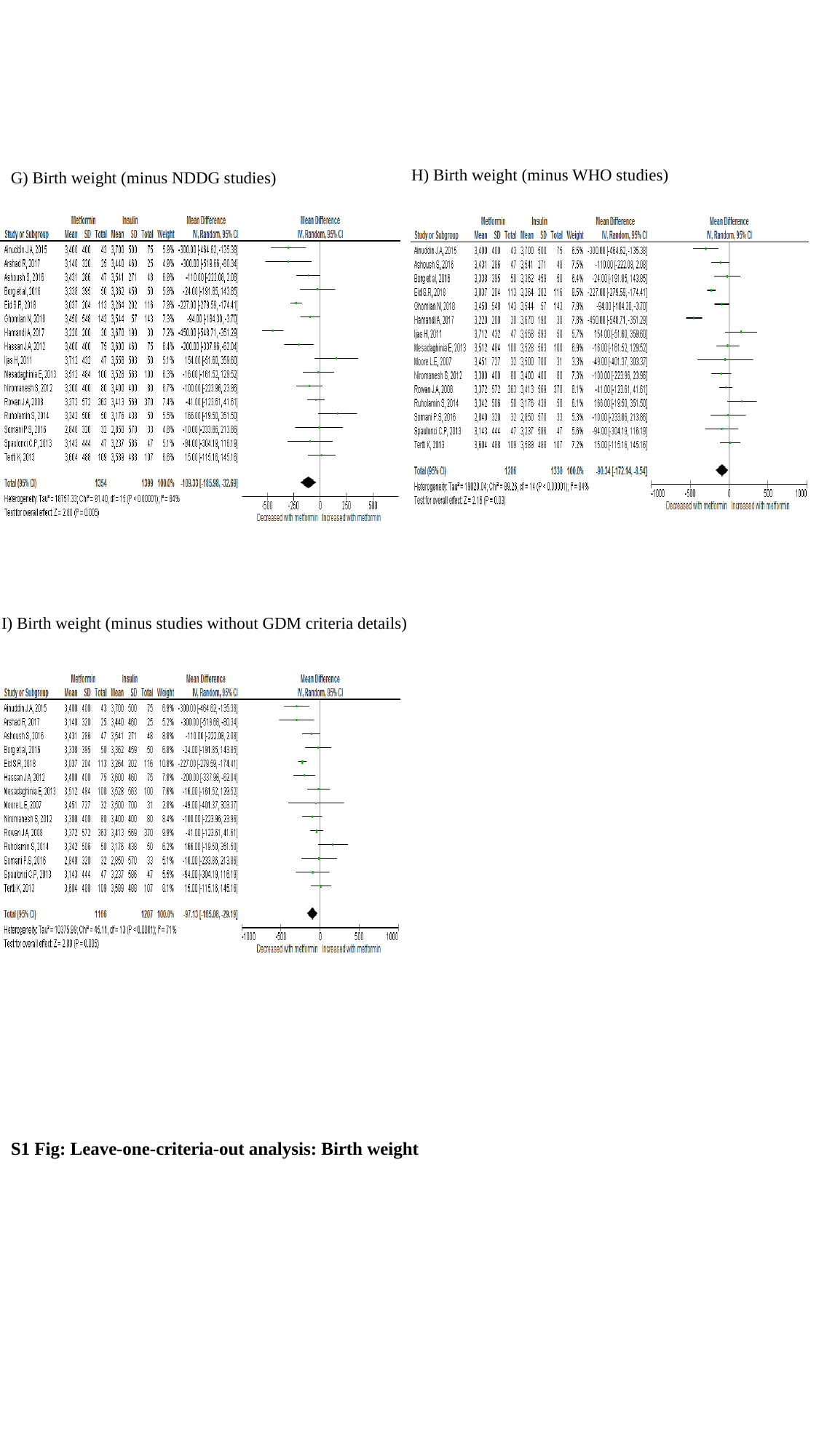

H) Birth weight (minus WHO studies)
G) Birth weight (minus NDDG studies)
I) Birth weight (minus studies without GDM criteria details)
S1 Fig: Leave-one-criteria-out analysis: Birth weight
