# Supplementary material for: Neonatal, infant, and childhood growth following metformin versus insulin treatment for gestational diabetes: A systematic review and meta-analysis
Source: PLoS Med. 2019 Aug 6;16(8):e1002848. doi: 10.1371/journal.pmed.1002848 (PMC6684046; doi:10.1371/journal.pmed.1002848)
Supplement: S2 Fig — (A) Birth weight, (B) macrosomia, (C) LGA, and (D) SGA. All outcomes expressed as OR (95% CI), with the exception of birth weight, which is expressed as mean difference (95% CI). (PPTX) [file pmed.1002848.s003.pptx]

## Slide 1
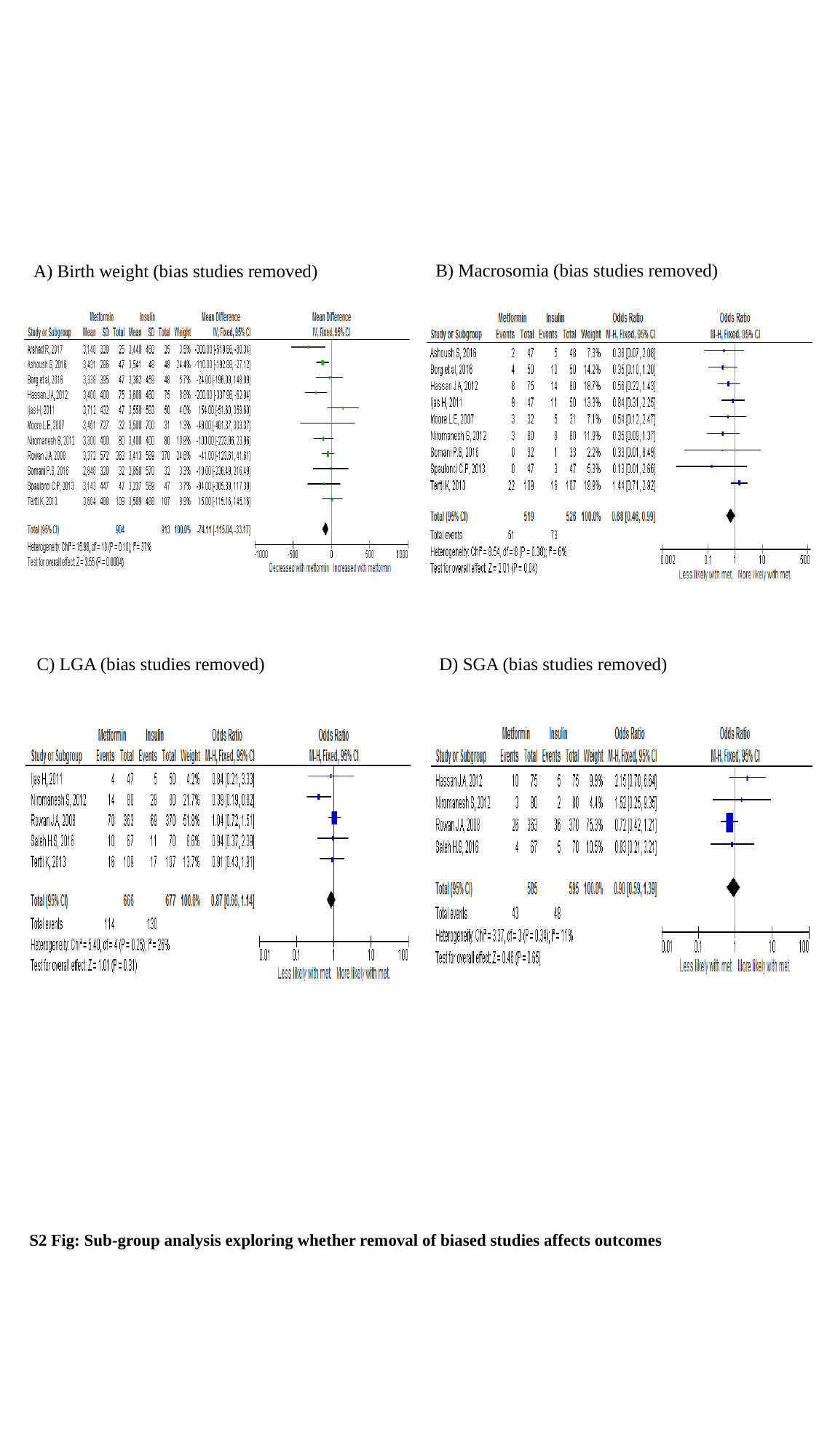

B) Macrosomia (bias studies removed)
A) Birth weight (bias studies removed)
C) LGA (bias studies removed)
D) SGA (bias studies removed)
S2 Fig: Sub-group analysis exploring whether removal of biased studies affects outcomes
