# Supplementary material for: Neonatal, infant, and childhood growth following metformin versus insulin treatment for gestational diabetes: A systematic review and meta-analysis
Source: PLoS Med. 2019 Aug 6;16(8):e1002848. doi: 10.1371/journal.pmed.1002848 (PMC6684046; doi:10.1371/journal.pmed.1002848)
Supplement: S3 Fig — (A) Birth weight, (B) macrosomia, (C) LGA, and (D) SGA. All outcomes expressed as OR (95% CI). (PPTX) [file pmed.1002848.s004.pptx]

## Slide 1
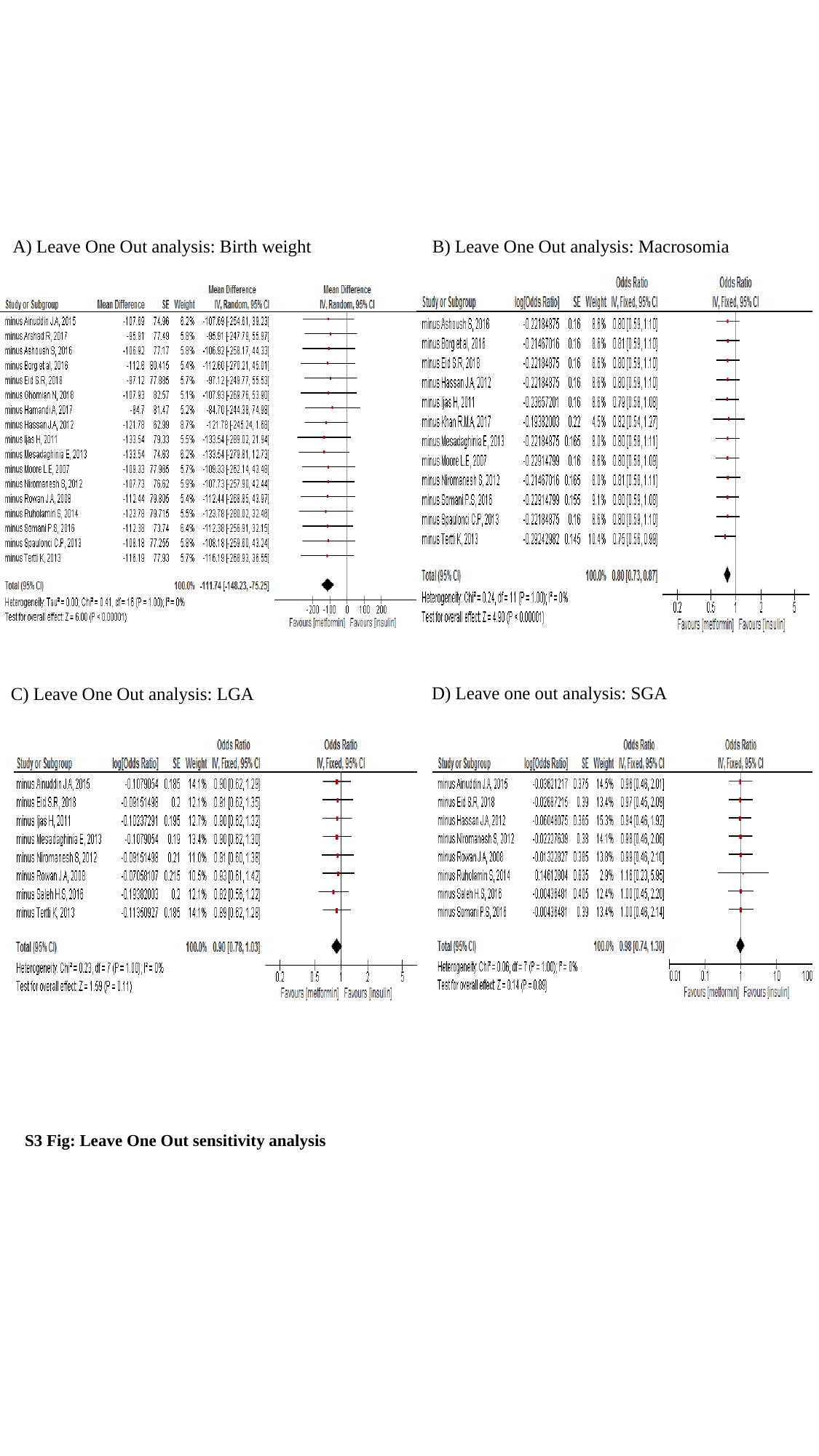

A) Leave One Out analysis: Birth weight
B) Leave One Out analysis: Macrosomia
D) Leave one out analysis: SGA
C) Leave One Out analysis: LGA
S3 Fig: Leave One Out sensitivity analysis
