# Supplementary material for: Neonatal, infant, and childhood growth following metformin versus insulin treatment for gestational diabetes: A systematic review and meta-analysis
Source: PLoS Med. 2019 Aug 6;16(8):e1002848. doi: 10.1371/journal.pmed.1002848 (PMC6684046; doi:10.1371/journal.pmed.1002848)
Supplement: S4 Fig — All outcomes plotted. (PPTX) [file pmed.1002848.s005.pptx]

## Slide 1
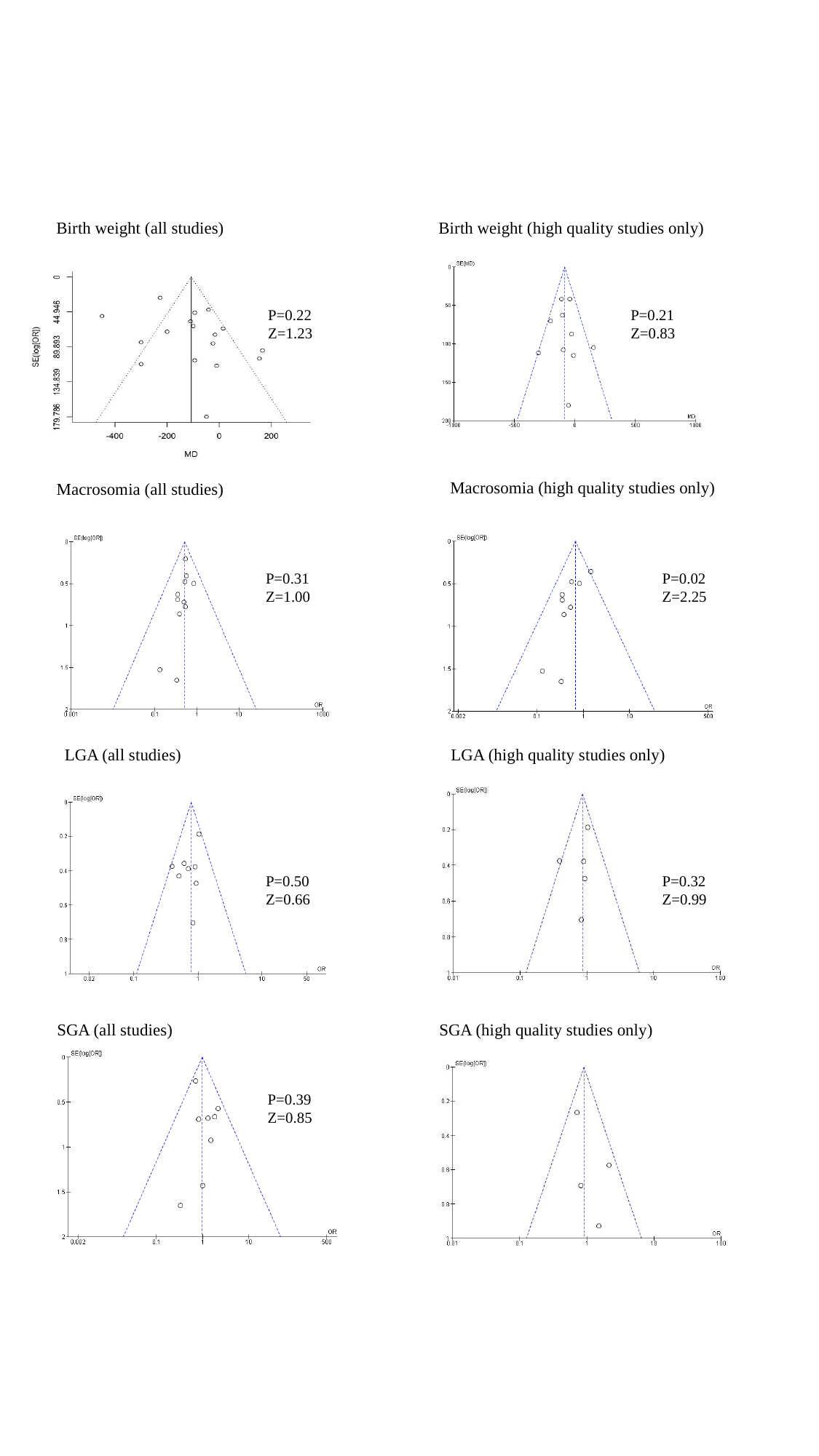

Birth weight (all studies)
Birth weight (high quality studies only)
P=0.22
Z=1.23
P=0.21
Z=0.83
Macrosomia (high quality studies only)
Macrosomia (all studies)
P=0.02
Z=2.25
P=0.31
Z=1.00
LGA (all studies)
LGA (high quality studies only)
P=0.50
Z=0.66
P=0.32
Z=0.99
SGA (all studies)
SGA (high quality studies only)
P=0.39
Z=0.85

## Slide 2
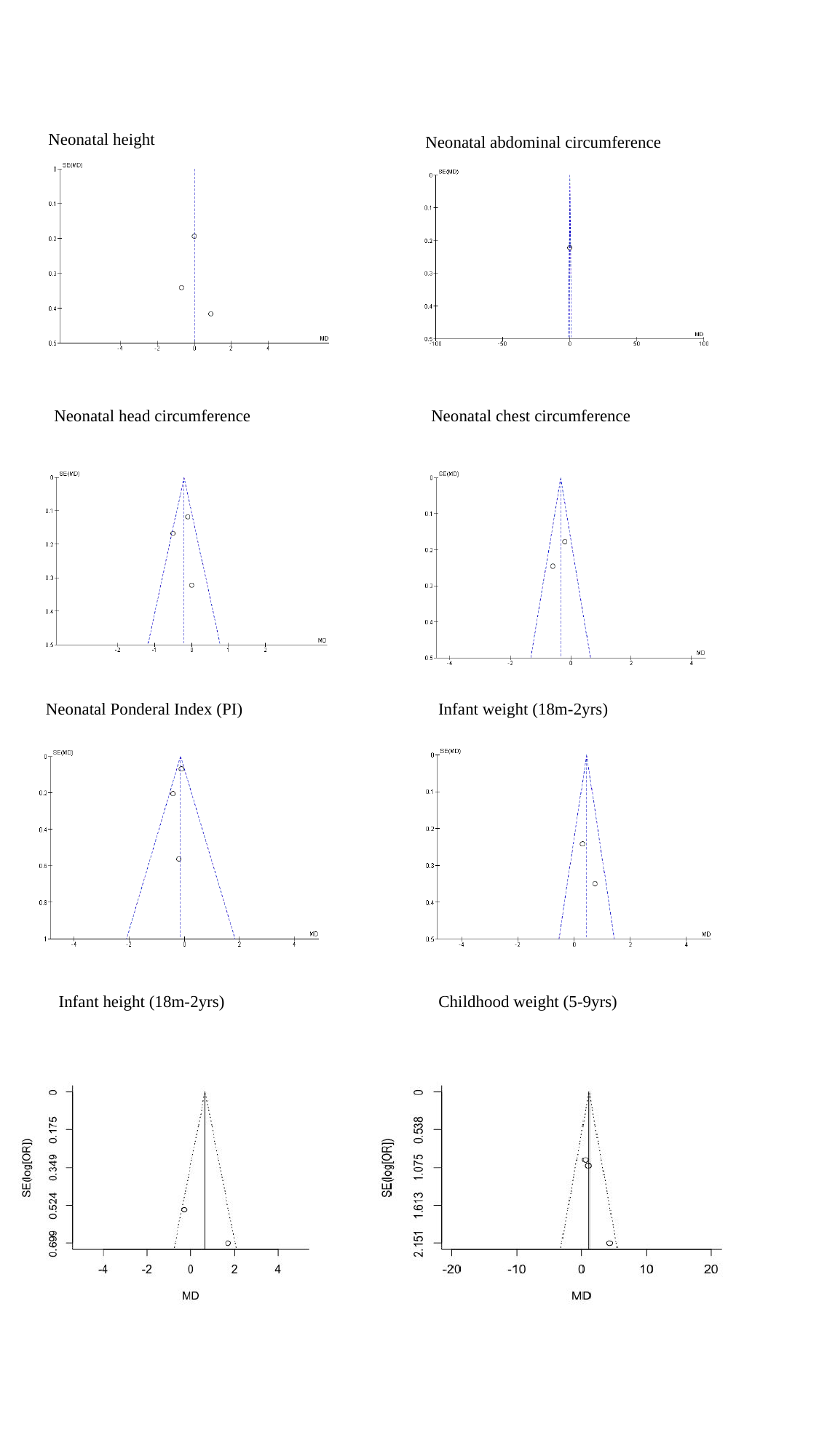

Neonatal height
Neonatal abdominal circumference
Neonatal head circumference
Neonatal chest circumference
Neonatal Ponderal Index (PI)
Infant weight (18m-2yrs)
Infant height (18m-2yrs)
Childhood weight (5-9yrs)

## Slide 3
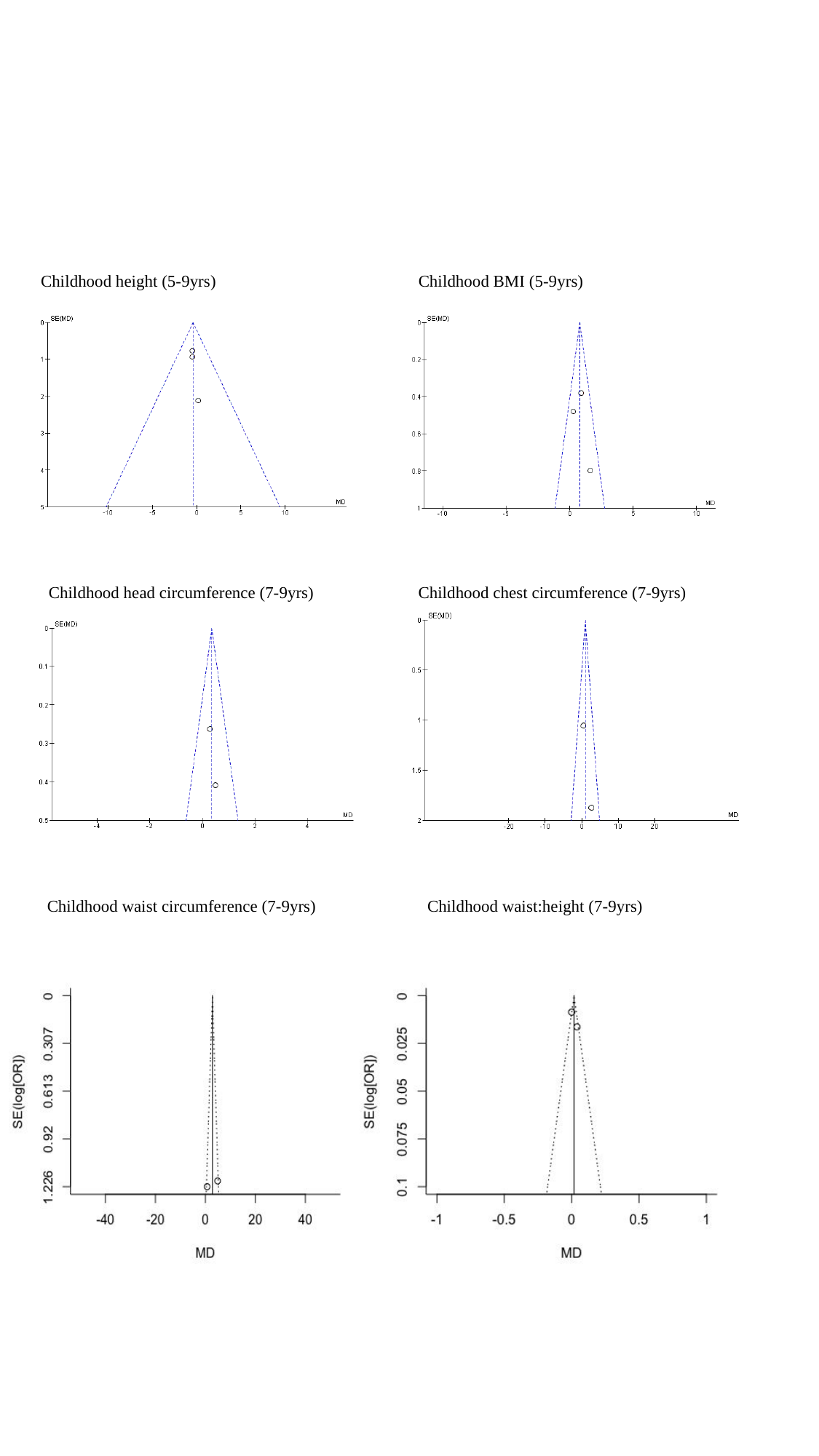

Childhood height (5-9yrs)
Childhood BMI (5-9yrs)
Childhood head circumference (7-9yrs)
Childhood chest circumference (7-9yrs)
Childhood waist circumference (7-9yrs)
Childhood waist:height (7-9yrs)

## Slide 4
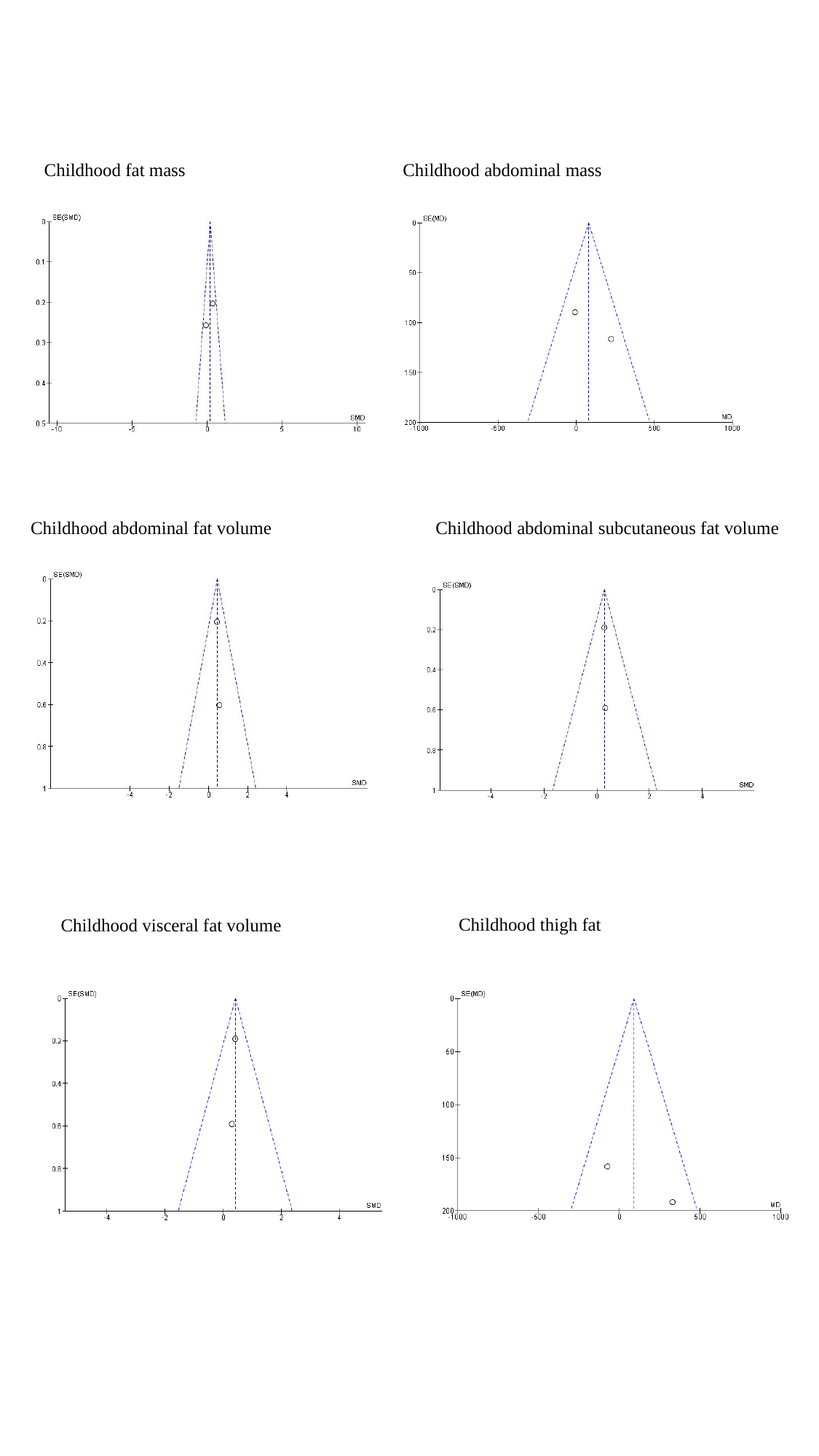

Childhood abdominal mass
Childhood fat mass
Childhood abdominal subcutaneous fat volume
Childhood abdominal fat volume
Childhood thigh fat
Childhood visceral fat volume

## Slide 5
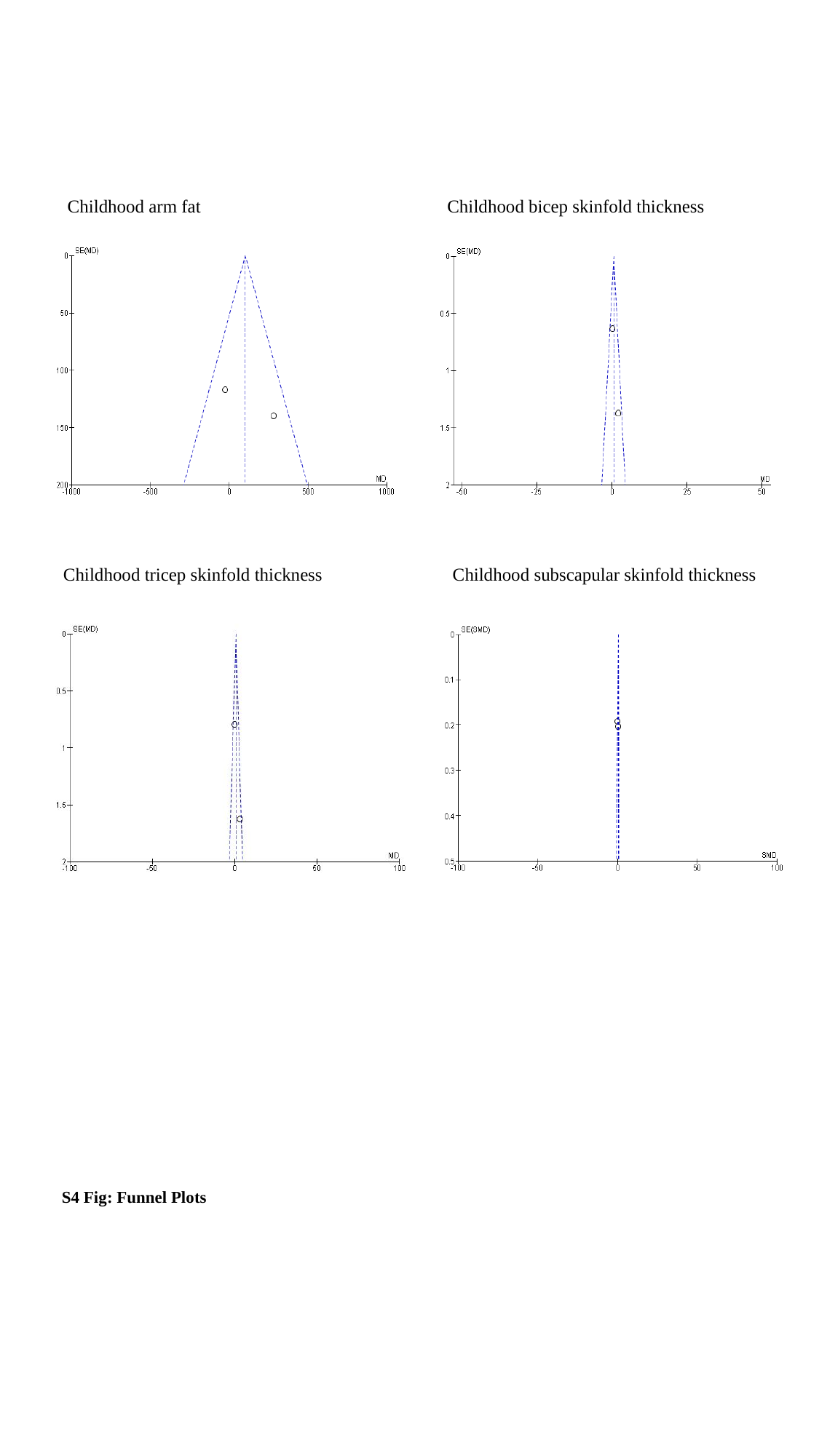

Childhood arm fat
Childhood bicep skinfold thickness
Childhood subscapular skinfold thickness
Childhood tricep skinfold thickness
S4 Fig: Funnel Plots
