# Supplementary material for: Neonatal, infant, and childhood growth following metformin versus insulin treatment for gestational diabetes: A systematic review and meta-analysis
Source: PLoS Med. 2019 Aug 6;16(8):e1002848. doi: 10.1371/journal.pmed.1002848 (PMC6684046; doi:10.1371/journal.pmed.1002848)
Supplement: S5 Fig — (A) Childhood head circumference, (B) childhood chest circumference, (C) childhood waist circumference, and (D) childhood waist:hip ratio. All outcomes expressed as mean differences (95% CI). (PPTX) [file pmed.1002848.s006.pptx]

## Slide 1
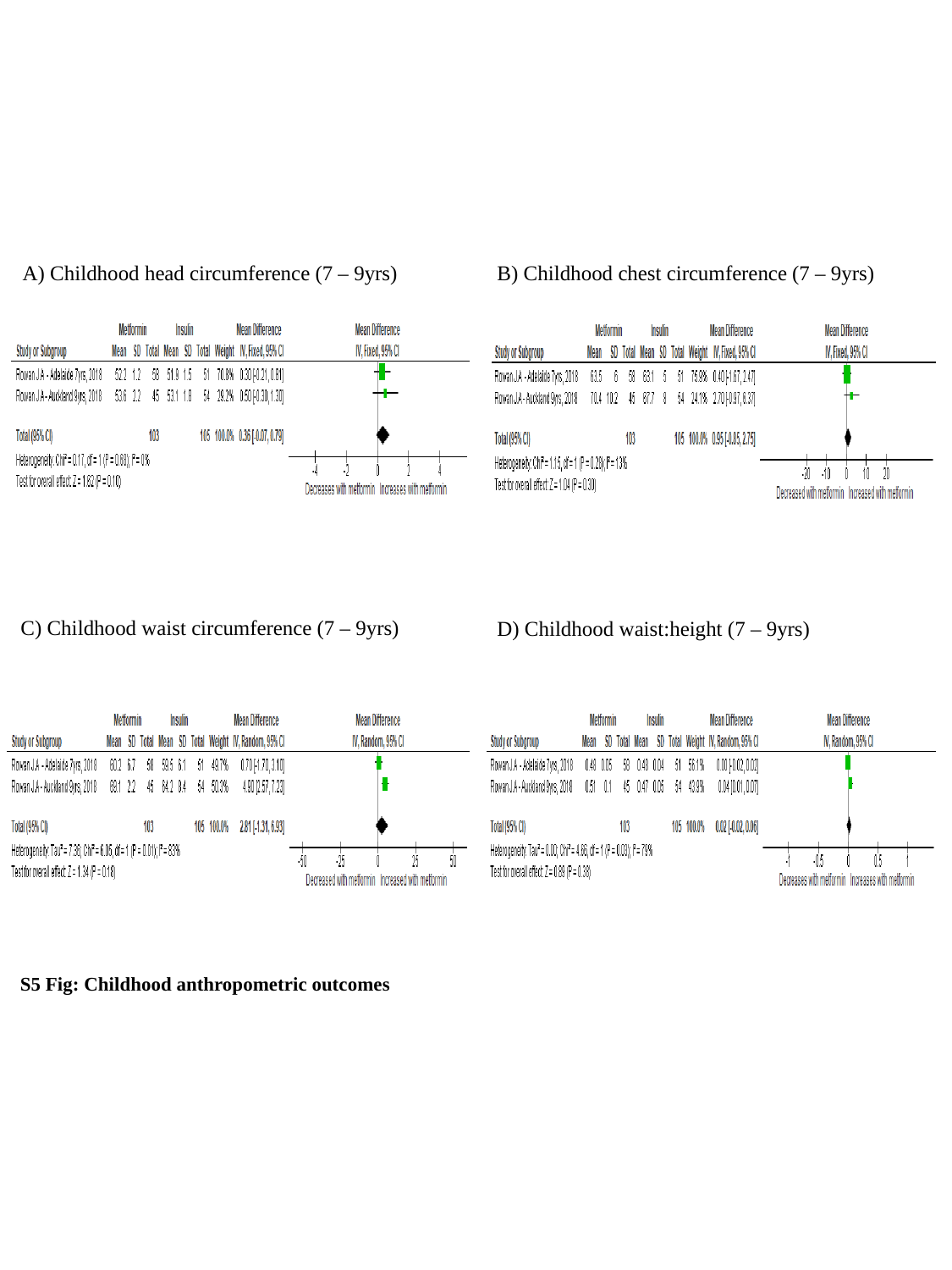

A) Childhood head circumference (7 – 9yrs)
B) Childhood chest circumference (7 – 9yrs)
C) Childhood waist circumference (7 – 9yrs)
D) Childhood waist:height (7 – 9yrs)
S5 Fig: Childhood anthropometric outcomes
