# Supplementary material for: Neonatal, infant, and childhood growth following metformin versus insulin treatment for gestational diabetes: A systematic review and meta-analysis
Source: PLoS Med. 2019 Aug 6;16(8):e1002848. doi: 10.1371/journal.pmed.1002848 (PMC6684046; doi:10.1371/journal.pmed.1002848)
Supplement: S1 Table — (PPTX) [file pmed.1002848.s007.pptx]

## Slide 1
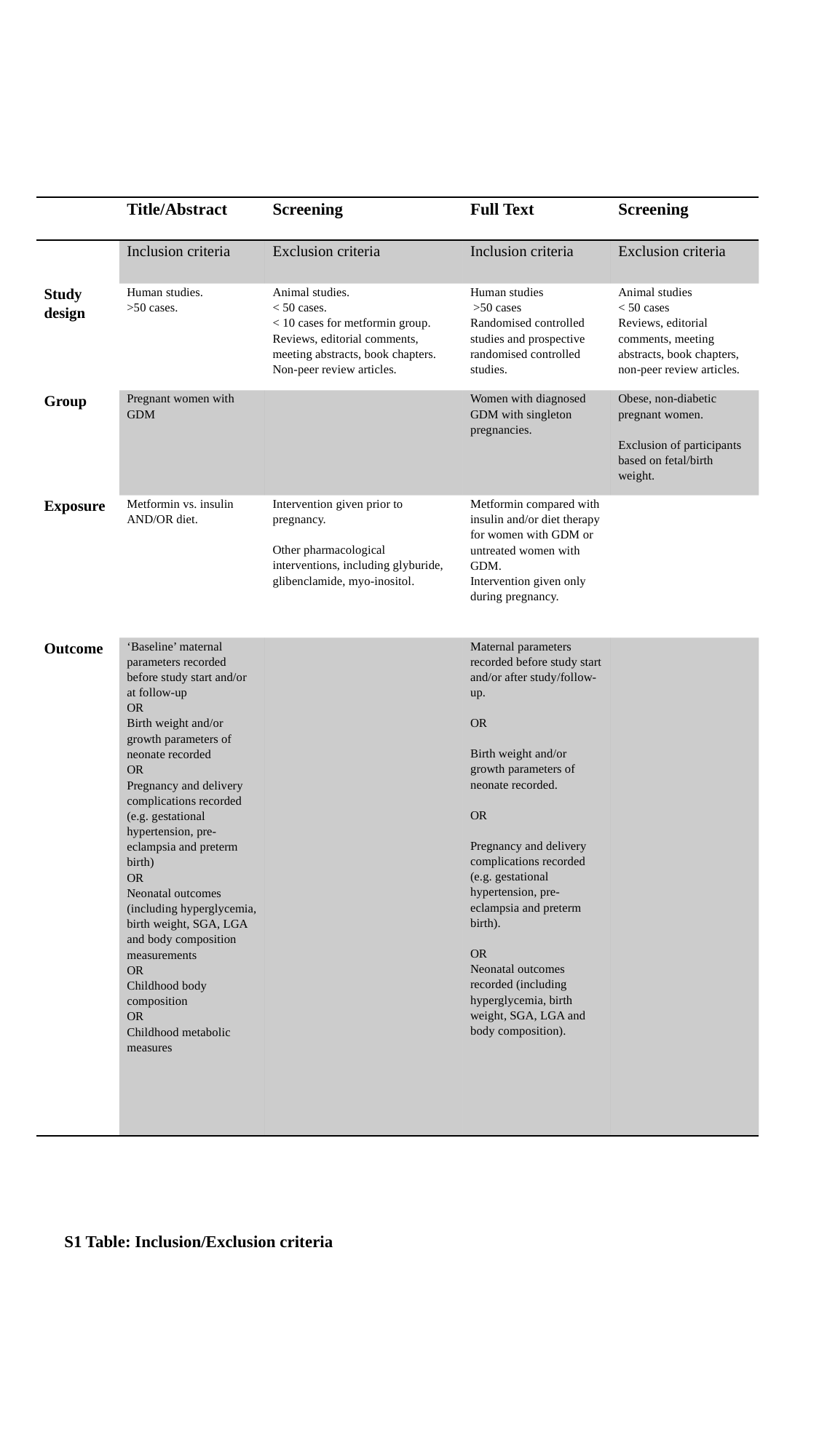

| | Title/Abstract | Screening | Full Text | Screening |
| --- | --- | --- | --- | --- |
| | Inclusion criteria | Exclusion criteria | Inclusion criteria | Exclusion criteria |
| Study design | Human studies. >50 cases. | Animal studies. < 50 cases. < 10 cases for metformin group. Reviews, editorial comments, meeting abstracts, book chapters. Non-peer review articles. | Human studies >50 cases Randomised controlled studies and prospective randomised controlled studies. | Animal studies < 50 cases Reviews, editorial comments, meeting abstracts, book chapters, non-peer review articles. |
| Group | Pregnant women with GDM | | Women with diagnosed GDM with singleton pregnancies. | Obese, non-diabetic pregnant women. Exclusion of participants based on fetal/birth weight. |
| Exposure | Metformin vs. insulin AND/OR diet. | Intervention given prior to pregnancy. Other pharmacological interventions, including glyburide, glibenclamide, myo-inositol. | Metformin compared with insulin and/or diet therapy for women with GDM or untreated women with GDM. Intervention given only during pregnancy. | |
| Outcome | ‘Baseline’ maternal parameters recorded before study start and/or at follow-up OR Birth weight and/or growth parameters of neonate recorded OR Pregnancy and delivery complications recorded (e.g. gestational hypertension, pre-eclampsia and preterm birth) OR Neonatal outcomes (including hyperglycemia, birth weight, SGA, LGA and body composition measurements OR Childhood body composition OR Childhood metabolic measures | | Maternal parameters recorded before study start and/or after study/follow-up. OR Birth weight and/or growth parameters of neonate recorded. OR Pregnancy and delivery complications recorded (e.g. gestational hypertension, pre-eclampsia and preterm birth). OR Neonatal outcomes recorded (including hyperglycemia, birth weight, SGA, LGA and body composition). | |
S1 Table: Inclusion/Exclusion criteria
