# Supplementary figures and images for: Neonatal, infant, and childhood growth following metformin versus insulin treatment for gestational diabetes: A systematic review and meta-analysis
Source: PLoS Med. 2019 Aug 6;16(8):e1002848. doi: 10.1371/journal.pmed.1002848 (PMC6684046; doi:10.1371/journal.pmed.1002848)

## Slide 1
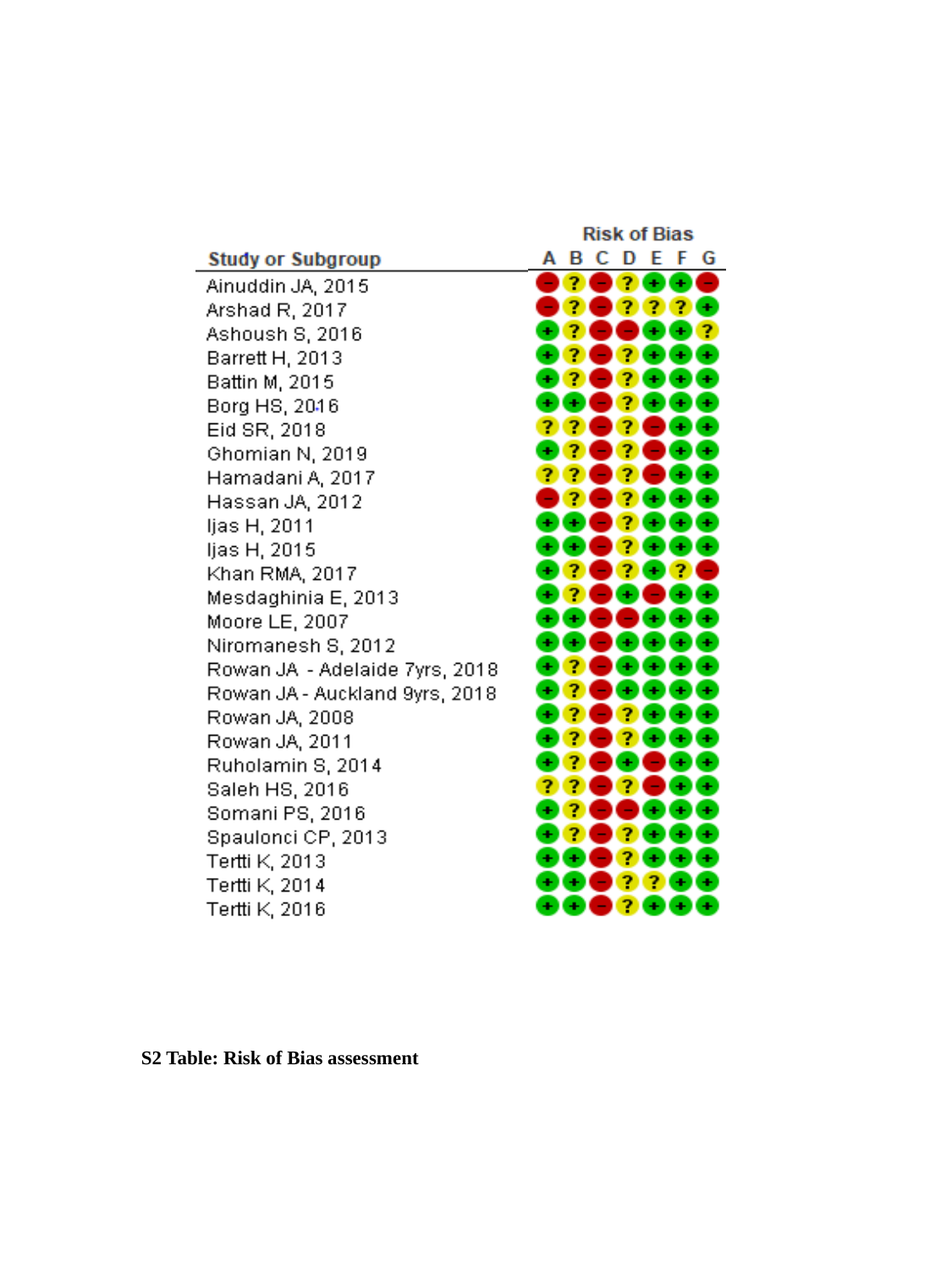

S2 Table: Risk of Bias assessment

Supplement: S2 Table — (A) Random sequence generation (selection bias), (B) allocation concealment (selection bias), (C) blinding participants and personnel (performance bias), (D) blinding of outcome assessment (detection bias), (E) incomplete oucome data (attrition bias), (F) selection bias (reporting bias), and (G) other bias. (PPTX) [file pmed.1002848.s008.pptx]

## Slide 1
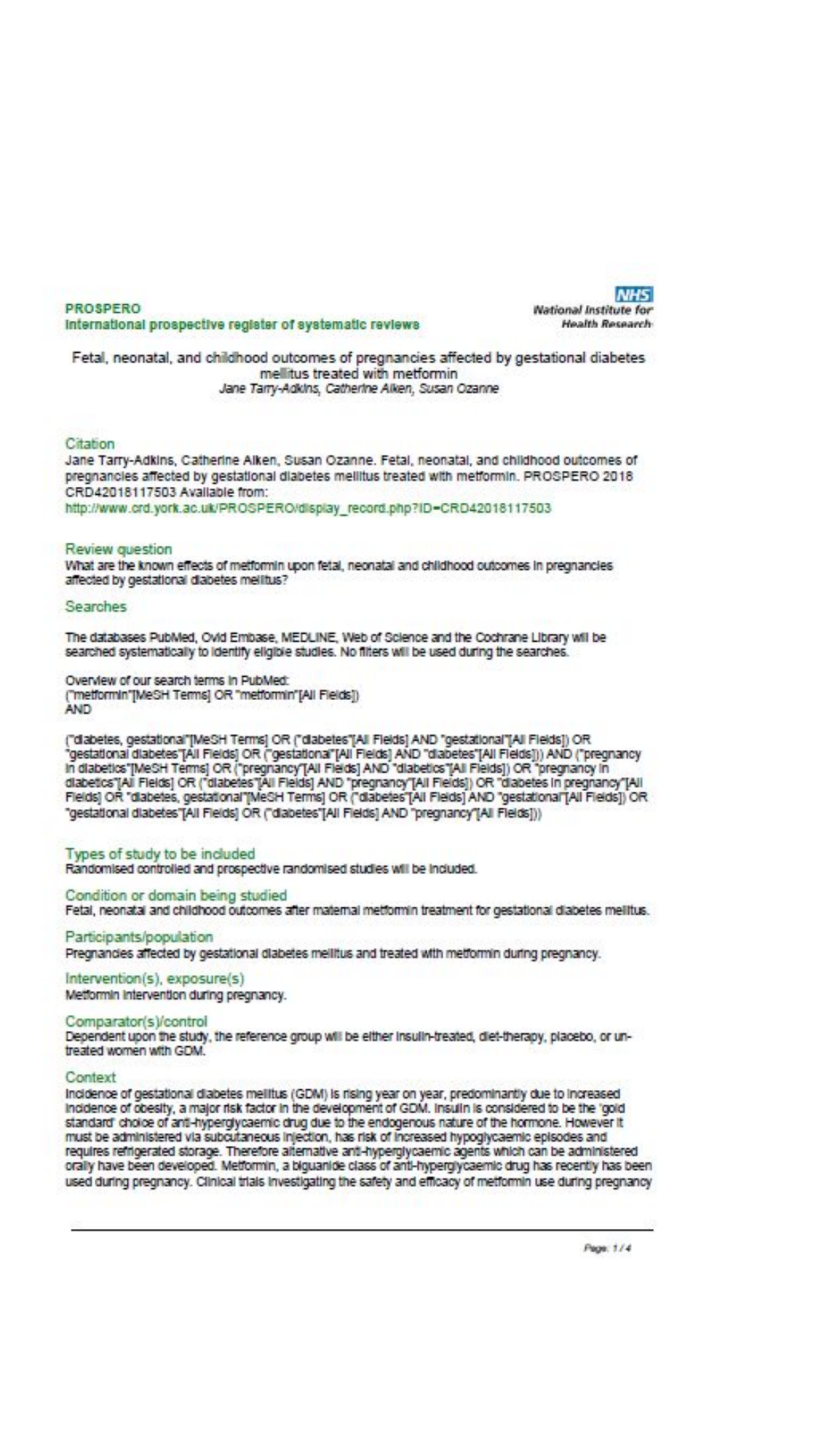

## Slide 2
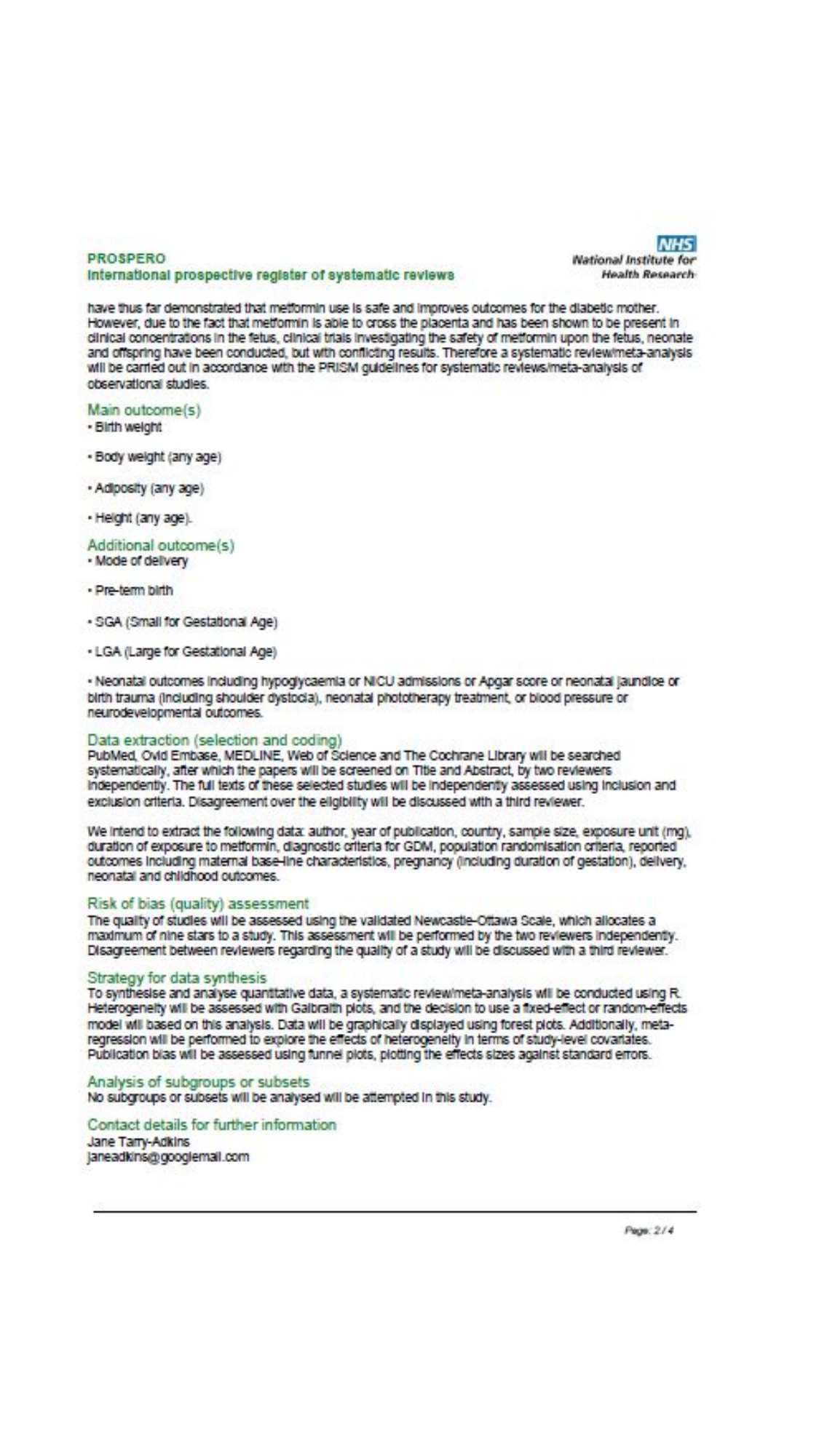

## Slide 3
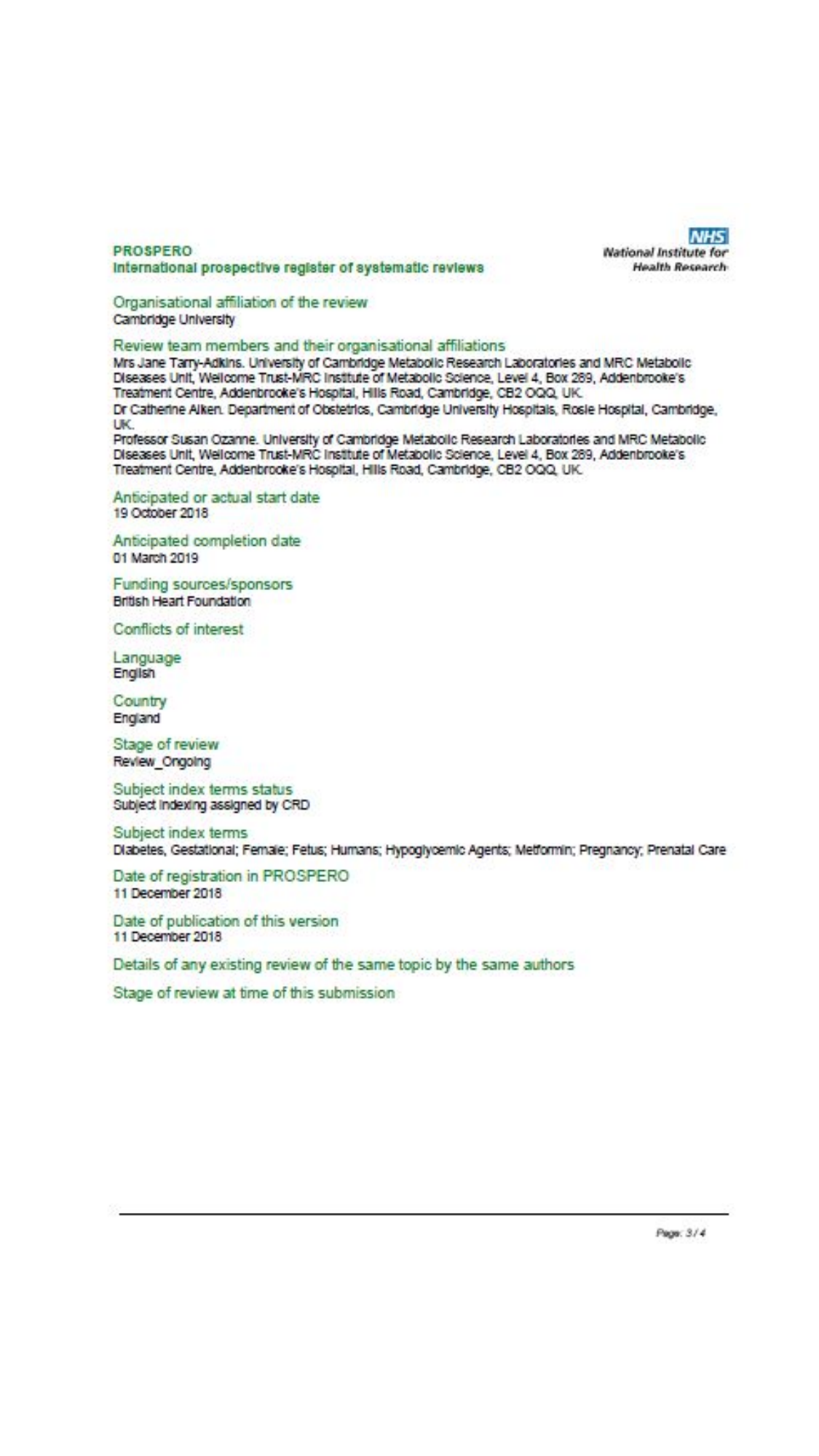

## Slide 4
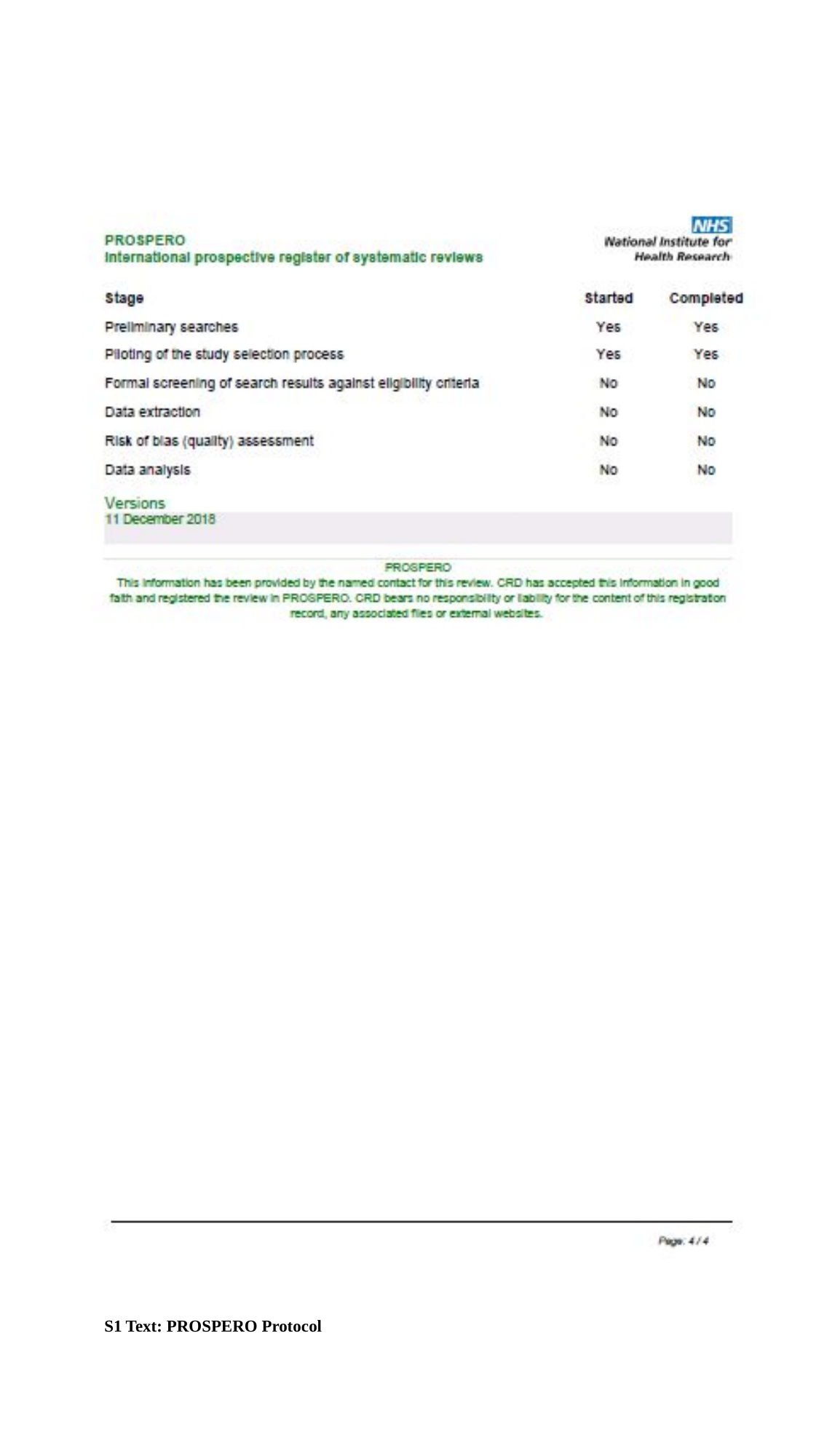

S1 Text: PROSPERO Protocol

Supplement: S1 Text — CRD42018117503. (PPTX) [file pmed.1002848.s010.pptx]
