# Supplementary material for: Neonatal, infant, and childhood growth following metformin versus insulin treatment for gestational diabetes: A systematic review and meta-analysis
Source: PLoS Med. 2019 Aug 6;16(8):e1002848. doi: 10.1371/journal.pmed.1002848 (PMC6684046; doi:10.1371/journal.pmed.1002848)
Supplement: S2 Text — (A) PubMed, (B) Ovid Embase, (C) Medline, (D) Web of Science, (E) Cochrane Library, and (F) ClinTrials.gov. (PPTX) [file pmed.1002848.s011.pptx]

## Slide 1
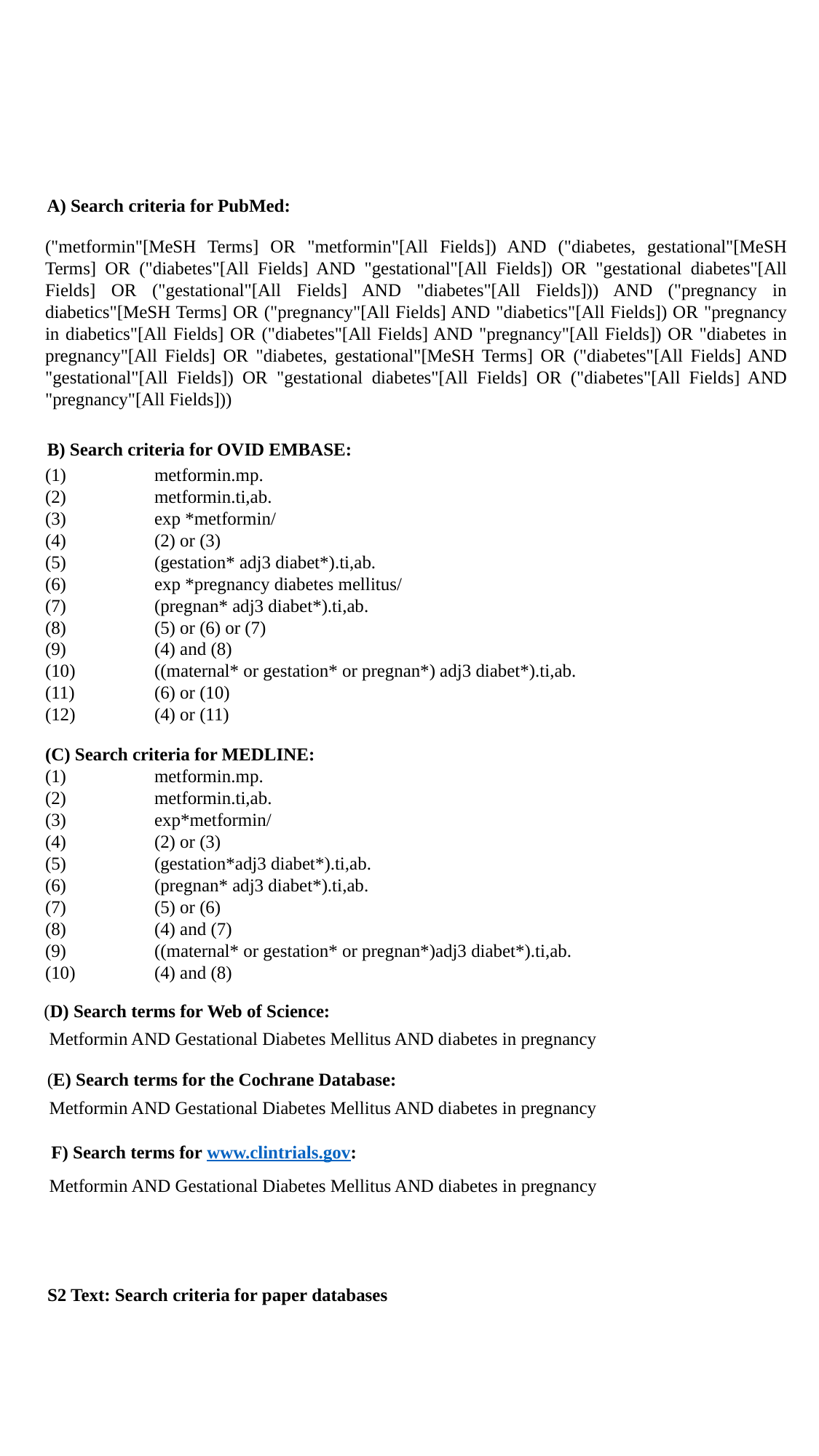

A) Search criteria for PubMed:
("metformin"[MeSH Terms] OR "metformin"[All Fields]) AND ("diabetes, gestational"[MeSH Terms] OR ("diabetes"[All Fields] AND "gestational"[All Fields]) OR "gestational diabetes"[All Fields] OR ("gestational"[All Fields] AND "diabetes"[All Fields])) AND ("pregnancy in diabetics"[MeSH Terms] OR ("pregnancy"[All Fields] AND "diabetics"[All Fields]) OR "pregnancy in diabetics"[All Fields] OR ("diabetes"[All Fields] AND "pregnancy"[All Fields]) OR "diabetes in pregnancy"[All Fields] OR "diabetes, gestational"[MeSH Terms] OR ("diabetes"[All Fields] AND "gestational"[All Fields]) OR "gestational diabetes"[All Fields] OR ("diabetes"[All Fields] AND "pregnancy"[All Fields]))
B) Search criteria for OVID EMBASE:
(1)	metformin.mp.
(2)	metformin.ti,ab.
(3)	exp *metformin/
(4)	(2) or (3)
(5)	(gestation* adj3 diabet*).ti,ab.
(6)	exp *pregnancy diabetes mellitus/
(7)	(pregnan* adj3 diabet*).ti,ab.
(8)	(5) or (6) or (7)
(9) 	(4) and (8)
(10) 	((maternal* or gestation* or pregnan*) adj3 diabet*).ti,ab.
(11) 	(6) or (10)
(12) 	(4) or (11)
(C) Search criteria for MEDLINE:
(1) 	metformin.mp.
(2) 	metformin.ti,ab.
(3)	exp*metformin/
(4)	(2) or (3)
(5)	(gestation*adj3 diabet*).ti,ab.
(6)	(pregnan* adj3 diabet*).ti,ab.
(7)	(5) or (6)
(8)	(4) and (7)
(9)	((maternal* or gestation* or pregnan*)adj3 diabet*).ti,ab.
(10)	(4) and (8)
(D) Search terms for Web of Science:
Metformin AND Gestational Diabetes Mellitus AND diabetes in pregnancy
(E) Search terms for the Cochrane Database:
Metformin AND Gestational Diabetes Mellitus AND diabetes in pregnancy
F) Search terms for www.clintrials.gov:
Metformin AND Gestational Diabetes Mellitus AND diabetes in pregnancy
S2 Text: Search criteria for paper databases
